# Fuzzy based residual shufflenet based breast cancer detection using mammogram images

**DOI:** 10.1038/s41598-026-63858-5

**Published:** 2026-08-03

**Authors:** Kumari Jelli, Pavan Kumar Pagadala

**Affiliations:** Department of Computer Science and Engineering, Koneru Lakshmaiah Education Foundation, Hyderabad, Telangana 500075 India

**Keywords:** Mammography, Computer-aided diagnosis, ShuffleNet, Deep learning, Breast cancer, Cancer, Computational biology and bioinformatics, Engineering, Mathematics and computing

## Abstract

Breast cancer is a condition in which abnormal breast cells grow and divide uncontrollably, resulting in the development of cancerous tissue. Detecting breast cancer at the preliminary phase is vital for improving survival rates and diagnostic outcomes. Though numerous Deep Learning (DL) frameworks have been proposed for detecting breast cancer, attaining accurate outcomes remains challenging. Thus, the Fuzzy-based Residual-ShuffleNet (Fuzzy RS-Net) is proposed for detecting breast cancer using mammogram images. Primarily, the mammogram image sourced from the dataset is fed as input to the wavelet domain filtering for noise reduction in the pre-processing stage. Further, the Object Segmentation Network (O-SegNet) is employed to segregate the affected region. Afterwards, image augmentation is effectuated using processes, such as rotation, shifting, and erasing. Further, feature extraction is processed, where texture features, Pyramid Histogram of Oriented Gradients (PHOG), and Weber Local Binary Pattern (WLBP) are extracted. At last, Fuzzy RS-Net is exploited for detecting breast cancer. In addition, the Fuzzy RS-Net is formulated by merging the Deep Residual Network (DRN), the Fuzzy concept, and ShuffleNet. Moreover, the developed Fuzzy RS-Net recorded the highest value of accuracy at 94.999%, sensitivity at 95.899%, and specificity at 93.889%.

## Introduction

Globally, non-communicable diseases (NCDs) are the primary cause of mortality. Moreover, cancer is considered the main contributor to a high mortality rate and is deemed a vital obstacle in revealing the life expectancy of people around the world^1^. The gradual expansion and development of abnormal cells in the human body is defined as cancer or tumor^2^. In 2018, the number of women reported to have breast cancer across the world exceeded 2.1 million. In addition, Breast cancer is determined as the most common malignancy, which causes a high death rate in women when compared with all other tumors^1^. It occurs when irregular cell growth leads to tumor formation, often noticeable as a lump or visible on an X-ray. The most dangerous type of tumor caused in women is Breast cancer, which affects both life and health of the people and is ranked first among all female diseases, and this cancer is considered the second most common spreading in women^3,4^. The main signs of breast cancer include abnormalities, namely changes in breast skin color, breast pain, formation of a breast mass, and changes in size and shape^5^. According to the statistics of the World Health Organization (WHO) in the year 2020, more than 2.3 million women were diagnosed with breast cancer^6^. WHO has reported that more than 685,000 deaths among women are attributed to breast cancer worldwide by the end of 2020^7^.

It is widely recognized that preventing breast cancer is extremely challenging. Nevertheless, the efficient diagnosis of breast cancer at the early stages can improve the recovery rate^1^. Early detection of breast cancer is essential for accurate assessment and diagnosis, and many researchers have adopted biomedical imaging approaches to assist expert radiologists^8^. In the current situation, computed tomography, mammography, Magnetic resonance imaging (MRI), microwave imaging, and photoacoustic imaging are employed for breast cancer identification^3^^9^. Radiotherapists widely use mammography for screening and diagnosing breast cancer. This method involves low-dose X-rays that allow clear imaging of the breast’s internal tissues. Usually, mammography is utilized for identifying approximately 80–90% of breast cancer that occurs in females^10^. However, specialist radiologists are still unable to detect the abnormalities during the initial phase of cancer. Detecting doubtful areas in X-ray images is considered a significant difficulty for radiologists. By identifying cancer before symptoms appear, mammography supports early diagnosis and reduces the risk of the disease advancing. Recent developments in mammography consider computer-aided detection, digital mammography, and breast tomosynthesis^1^. Simultaneously, the abnormal condition, namely masses and calcification, and other subtle symptoms such as bilateral asymmetry and architectural distortion can be identified by utilizing the mammography^5^.

In addition, manually interpreting medical images in a large volume requires sufficient time and it is considered a monotonous process^11^. Moreover, the responsibilities of radiologists can be reduced with Computer-Aided Diagnosis (CAD) systems, which are supported by Artificial Intelligence (AI) methods for identifying malignant tissues and cancer^1^^12^. Various types of image-processing approaches, like Machine Learning (ML) methods including Naïve Bayesian (NB), Artificial Neural Networks (ANN), Support Vector Machine (SVM), and Convolution Neural Networks (CNN), are exploited for identifying breast cancer^7^. Variability in texture, size, position, and shape makes lesion detection a challenging task. In addition, image processing and Deep Learning (DL) techniques have been developed to overcome the challenges of conventional Techniques, which lack the capability of automated detection^8^^9^. Moreover, DL is utilized in medical imaging to improve automated CAD systems and is regarded as the most efficient approach for identifying and classifying medical images^8,13^. Recently, numerous DL-based CAD systems have been utilized by radiologists for the process of screening, diagnosis, and monitoring breast cancer. Current research has recommended the use of DL-based segmentation models and object detection approaches for diagnosing breast cancer due to their reliability and accuracy. Still, before implementing these approaches to identify breast cancer, particularly when utilizing high-resolution digital mammography images, various difficulties need to be considered carefully^8^^12^^14^. Moreover, several DL models, such as hybrid rule-based, Deep Convolutional Neural Network (DCNN), deep fuzzy model, and so on, have been proposed for diagnosing breast cancer at the early stage^15^^14^.

DL-based and hybrid breast cancer detection approaches are challenged by high computational requirements, insufficient handling of uncertainty during mammogram analysis, poor discrimination of important features, and limited robustness when identifying subtle abnormalities in breast tissue. Fuzzy RS-Net, a novel framework, is proposed to improve breast cancer detection and overcome existing limitations. Unlike existing hybrid DL models that typically integrate either CNN–fuzzy or CNN–residual combinations, the proposed Fuzzy RS-Net introduces a unified three-way fusion of DRN, ShuffleNet, and fuzzy logic within a single framework. The process begins by loading the mammogram image from the dataset, followed by pre-processing with wavelet domain filtering. In the segmentation phase, O-SegNet is employed to isolate the affected areas in the image. Subsequently, image augmentation is performed using methods like rotation, shifting, and erasing. Thereafter, features, namely PHOG, WLBP, and texture features, namely Angular Second Moment (ASM), Sum Entropy, Maximal Correlation Coefficient, and Contrast, are excerpted. Lastly, the Fuzzy RS-Net model is established for breast cancer identification, integrating the Fuzzy concept, DRN, and ShuffleNet. The framework improves classification outcomes by integrating DRN for feature enhancement, ShuffleNet to minimize computational demands, and fuzzy logic to address uncertainty, thereby outperforming existing methods in accuracy, sensitivity, and robustness. By combining these components, the proposed design enhances feature representation, minimizes computational overhead, and efficiently handles uncertainty in mammogram interpretation, offering superior robustness and overall performance compared with previous approaches.

The primary contributions of this research are outlined as follows:

**Proposed fuzzy RS-net for breast cancer detection** An advanced Fuzzy RS-Net is presented in this research for breast cancer detection from mammograms, formed through the integration of fuzzy theory, ShuffleNet, and DRN.

The enduring segments of the work are systematized as ensuing. Section "Motivation" expounds on the available methodologies for diagnosing breast cancer. Section "Proposed method of fuzzy based residual-shufflenet (Fuzzy RS-Net) model for detection of breast cancer utilizing mammogram images." expounds on the devised technique; Section "Results and discussion" expounds on the experimental discussion, and Section "Conclusion" presents the conclusion.

## Motivation

This aggressive disease significantly impacts both the health and well-being of women. Detecting breast cancer from mammographic images is difficult because abnormal cells develop within the breast tissue, making accurate identification more complex. In addition, existing approaches for identifying breast cancer depend on manual feature extraction from mammograms and require expert-level medical knowledge. Accordingly, a novel Fuzzy RS-Net model is introduced in this study to improve breast cancer detection.

### Literature review

Ibrokhimov, B. and Kang, J.Y^6^ introduced a Two-stage deep learning framework for detecting breast cancer by utilizing high-resolution mammogram images. The proposed framework minimizes false positives and delivers improved reliability. However, this technique does not develop standalone classifiers to experiment with different network architectures for improving detection performance. Patil, R.S. and Biradar, N^3^ established a Firefly updated Chicken Swarm Optimization (FC-CSO) with a hybrid CNN + Recurrent Neural Network (RNN) classifier for automated mammogram-based breast cancer detection. This framework obtained a high convergence rate. Still, this technique did not include information on the thin lines and blurred images at low noise densities for enhancing the process of breast cancer recognition. Kavitha, T. et al.^10^ presented an Optimal Multi-Level Thresholding-based Segmentation-Deep Learning enabled Capsule Network (OMLTS-DLCN) to detect breast cancer. Although the framework successfully identified breast cancer class labels, it lacked an evaluation of hyperparameter tuning methods that could enhance the accuracy of detection and classification. Zebari, D.A., et al.^1^ devised a technique using Multi-Fractal Dimension (M-FD) for breast cancer detection. The proposed approach achieved early malignancy detection while using a smaller volume of information for model training. Nevertheless, the method failed to consider deep learning-based techniques for minimizing the processing time required for breast cancer detection.

Chouhan, N., et al.^5^ designed the Diverse Features-based Breast Cancer Detection (DFeBCD) system for detecting breast cancer. The approach achieved reliable breast cancer identification with minimal errors, yet it has not been tested in real-time applications. Wisaeng, K^7^., proposed the K–means + + clustering based on Cuckoo Search Optimization (KM + + CSO) for breast cancer detection. This method demonstrated efficient breast cancer detection in low-intensity mammograms with low computational requirements, but it lacked evaluation on large datasets and did not explore fixed clustering methods for initial coarse detection. Maqsood, S., et al.^16^ developed a Transferable Texture CNN (TTCNN) for the classification and detection of breast cancer. With minimal execution time and excellent robustness, this method proved highly efficient. Nevertheless, this approach has not been applied to other biomedical imaging tasks, including the detection of brain tumors. Rahman, H., et al.^8^ devised the Deep CNN (DCNN) for diagnosing breast cancer. This method minimized the error rate in screening mammogram images when diagnosing breast cancer. Nevertheless, the method did not examine extensive breast cancer datasets from different age categories to facilitate early detection of the disease.

Raj Kumar Pattanaik., *et.al.*^17^ developed a novel hybrid model called DenseNet121 + Extreme Learning Machine (ELM) for classifying breast cancer from mammogram images. It required fewer iterations to converge and demonstrated consistently lower training loss. Although this model required significant computational resources due to DenseNet121’s deep architecture. Dr. P.R. Futane., *et.al.*^18^ introduced a new method called Attention ResNet Coupled Convolutional Neural Network (A2Res-CNN) for breast cancer detection using mammographic images. It enhanced feature extraction by focusing on both channel dependencies and positional dependencies. A2Res-CNN improves upon conventional CNNs by reducing overfitting; however, it continues to require extensive datasets and high computational power for training. Dilawar Shah., *et.al.*^19^ proposed a new dual-view deep learning model called EfficientViewNet to overcome false predictions of breast cancer. The method utilized both craniocaudal (CC) and mediolateral oblique (MLO) perspectives to achieve fewer false positives and false negatives than single-view approaches. However, it relied heavily on the Radiological Society of North America (RSNA) benchmark dataset. Variations in annotation quality, density, and lesion visibility across datasets may affect generalizability.

### Challenges

The key limitations of traditional approaches used for detecting breast cancer from mammographic images are presented as follows.

- The Two-stage deep learning method^6^ was used by radiologists in the real-time application for the reporting and screening of breast cancer, and yet, this technique suffered from misclassified intra-class scores.
- The DFeBCD approach utilized in^5^, effectively detected the chance of benign and identified the subtypes of breast cancer from the mammogram images. Still, texture features were overlooked in the process of detecting abnormalities effectively.
- The KM + + CSO method^7^ efficiently tracked the lesions on the mammogram surface for the reliable detection of breast cancer. Still, this technique required different algorithmic techniques to contrast with the ground truth images. Besides, the approach was assessed on a smaller dataset, limiting its generalization.
- The TTCNN technique^16^ reduced computational complexity problems and decreased memory usage while identifying the tumor correctly. However, this technique suffers from overfitting and performs poorly on images with background noise.
- The approaches employed for diagnosing breast cancer identified the abnormal growth of the tissues and improved the accuracy of the process of diagnosis utilizing mammogram images. Nevertheless, these techniques were typically very sensitive to noise and weak boundaries and caused high computational complexity.

## Proposed method of fuzzy based residual-shufflenet (Fuzzy RS-Net) model for detection of breast cancer utilizing mammogram images

**Fig. 1:**
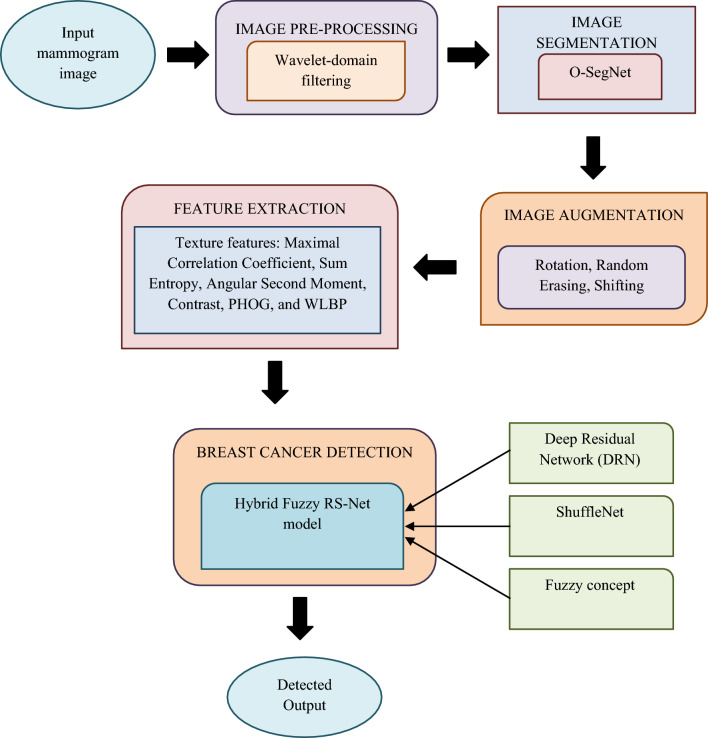
A diagrammatic view of the Fuzzy RS-Net for breast cancer by utilizing the mammogram image.

Considered a deadly illness among women, breast cancer is associated with high mortality and shortened lifespan. Detecting tumors early can greatly decrease the likelihood of death. Accordingly, Fuzzy RS-Net is developed as a new framework for mammogram-based breast cancer detection, beginning with the retrieval of the input image from a database. Afterwards, the image pre-processing is done by the Wavelet domain filtering^20^. Consequently, the segmentation is performed by using the O-SegNet^21^ for the process of segmenting the regions affected by breast cancer. Moreover, image augmentation is carried out by using processes, such as rotation, shifting, and erasing^22^. Subsequently, in the feature extraction process, features such as PHOG^23^, WLBP^24^, texture features^25^, namely Maximal Correlation Coefficient^26^, ASM^27^, Sum Entropy^28^, and Contrast^27^ are mined. In the final step, breast cancer is diagnosed through the Fuzzy RS-Net approach. The Fuzzy RS-Net is established by formulating the DRN^29^, ShuffleNet^30^, and the Fuzzy concept^31^, and the diagrammatic representation of the Fuzzy RS-Net for breast cancer by utilizing the mammogram image is shown in Fig. 1.

### Image collection

The mammographic dataset provides input images, labeled *S*, which are utilized for diagnosing breast cancer, as shown below,

1 $$S = \left\{ {S_{1} ,S_{2} ,.....S_{m} ,...S_{n} } \right\}$$

wherein,$S_{m}$ represents the $m^{th}$ image regarded for the detection process, and *n* indicates the overall count of images present in the dataset.

### Image pre-processing

To enhance the quality of the input images and minimize noise effects, a pre-processing technique is employed. Furthermore, the mammogram image $S_{m}$ is fed as the input to wavelet domain filtering^20^ for image preprocessing. The key notion behind this is to perform noise removal while preserving the visual perception of the image. Filtering is performed in three phases: applying the Discrete Wavelet Transform (DWT), filtering, and then applying the inverse DWT. DWT is mostly used in image processing due to its computational efficiency and multiresolution nature. It is utilized in the preprocessing stage due to its capability to perform both noise reduction and feature enhancement simultaneously. It effectively preserves important edge and texture information while suppressing noise, which is critical for accurate analysis of mammogram images. Unlike conventional sharpening techniques that may amplify noise and introduce artifacts, wavelet-based processing enhances significant structural details in a controlled manner. Therefore, additional sharpening or enhancement techniques are not applied, as the wavelet transform sufficiently improves image quality for subsequent processing stages. Here, the image is decomposed into a group of basic functions as follows,

The discrete wavelet at level $p^{\prime}$ are easily adapted to the 2D case for image preprocessing.

2 $$\hbar \left( {2^{e} V^{\prime}} \right) = \sum\nolimits_{{X^{\prime}}} {N^{\prime}_{{p^{\prime} + 1}} } \left( {X^{\prime}} \right)\ell \left( {2^{{p^{\prime} + 1}} V^{\prime} - X^{\prime}} \right)$$

where, $\ell \left( \right)$ denotes the scaling function, $V^{\prime}\,$ and $X^{\prime}$ are integers and $N^{\prime}$ indicates the wavelet filter.

The forward DWT of an image is attained by employing a filter bank with low-pass and high-pass filters, and then downscale sampling is applied. In this way, the image is split into various smaller detail images and approximation images. Detail coefficients are obtained using a high-pass filter, whereas a low-pass filter yields the approximation coefficients. Thereafter, thresholding filtering is applied to maintain the high-amplitude coefficients, which hold the main information about the images and avoid the approximation coefficients. The thresholding filter is executed using soft and hard thresholding.

The image is degraded by the additive white Gaussian noise of the variance $\sigma^{2}$ and zero mean in the wavelet domain, and it can be represented as follows,

3 $$D\left( {d,e} \right) = E\left( {d,e} \right) + F\left( {d,e} \right)$$

here,$D\left( {d,e} \right)$ denotes the noisy images,$F\left( {d,e} \right)$ embodies the additive white Gaussian noise, and $E\left( {d,e} \right)$ indicates the noiseless images,

Furthermore, the soft and hard thresholding with a threshold equal to the *℘* are expressed in the (3) and (4) equations. The soft thresholding is expressed as,

4 $$\mathop {\hat{D}_{\wp }^{Soft}} = \left\{ {\begin{array}{*{20}c} {\left( {sgn\left( {D\left( {d,e} \right)} \right)\left( {\left| {D\left( {d,e} \right)} \right|} \right) - \wp } \right)} & {\left| {D\left( {d,e} \right)} \right| \succ \wp } \\ 0 & {\left| {D\left( {d,e} \right)} \right| \ge \wp } \\ \end{array} } \right\}$$

here,*℘* indicates the threshold value.

The hard thresholding is characterized as,

5 $$\mathop {\hat{D}_{\wp }^{Hard}} = \left\{ {\begin{array}{*{20}c} {D\left( {d,e} \right)} & {\left| {D\left( {d,e} \right)} \right| \succ \wp } \\ 0 & {\left| {D\left( {d,e} \right)} \right| \ge \wp } \\ \end{array} } \right\}$$

In addition, the threshold *℘* is formulated by means of the Sum of Absolute Differences (SAD) value in each matched block. The threshold for the matching block is formulated as,

6 $$\wp_{soft} = average\left( {\wp_{1} ,....,\wp_{f} ,\wp_{block} ,} \right)f = 1.....32$$

The wavelet filtering technique’s last step is the inverse discrete transform. Moreover, wavelet filtering is utilized for noise reduction in the referenced block, and the image preprocessing output is indicated as $S_{m}^{pre}$.

### Cancer region segmentation

This process signifies the task of detecting and segregating the affected regions in the mammogram image. This process is imperative as it provides information about the size and shape of the tumor, thus enhancing the detection process. The pre-processed image $S_{m}^{pre}$ is fed to the O-SegNet for segmenting the cancer region.

#### Architecture of O-SegNet

The O-SegNet^21^ is comprised of the reconstruction stages (decoder), context aggregation, and feature learning (encoder), for the process of deriving the differently shaped areas in the pre-processed image $S_{m}^{pre}$. O-SegNet is mainly used here due to its robustness, generalization ability, and high segmentation accuracy. The proposed feature learning architecture integrates convolutional blocks, GA blocks, and max-pooling layers, where the GA block takes the intermediate features learned from two preceding convolutional block groups as input. A Self-Attention (SA) module is also applied to integrate attention mechanisms into the features. The attentive features, reduced by a factor of two in dimensionality, are passed to the subsequent GA block, with this procedure repeated for the remaining two resolutions. By combining earlier learned features with attention-aware features, the encoder creates discriminative feature maps, and GA blocks are further applied to model pixel relationships. In addition, encoder-extracted features are input to an 8-level Pyramid Pooling Network (PPN), where feature aggregation is carried out across multiple depth levels.

**Fig. 2:**
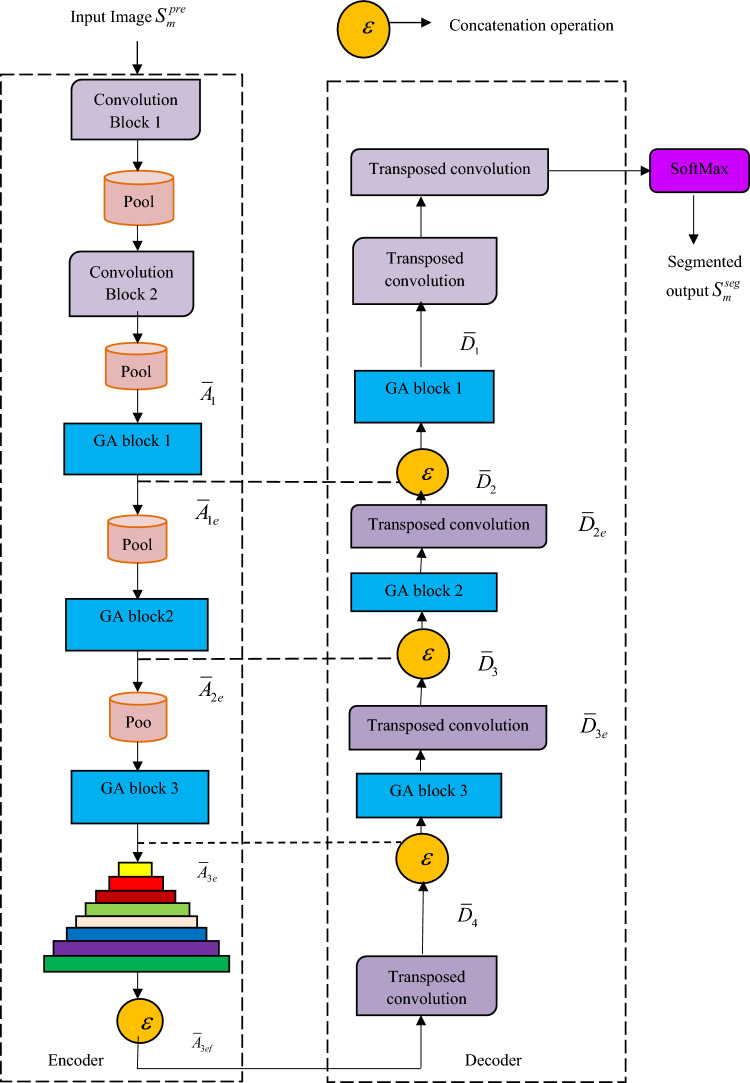
An outline for O-SegNet.

The integration of multiple encoder feature variants enables global feature extraction. In the reconstruction stage, the concatenated outputs of the 8-level PPN are forwarded through transposed convolution layers to restore the initial resolution using the compressed features generated by the encoder. The reconstruction process employs transposed convolutions to maintain the encoder-derived features at every stage, after which the obtained features are forwarded to the GA blocks. Consequently, the up-sampled and encoder features incorporate attention mechanisms. The decoder GA block integrates attentive-aware features with prior upsampled features, applying the same process across the remaining resolutions. The decoder produces a precise segmentation map from the encoder features through the application of attention-guided upsampling and downsampling processes. The segmented output attained is depicted as $S_{m}^{seg}$. An outline of O-SegNet is exemplified in Fig. 2.

### Image augmentation

This process is mostly used in computer vision and ML approaches for expanding the dimension of the training data. By subjecting the original images to various transformations, this method promotes improved generalization. The image augmentation process is applied to the segmented image $S_{m}^{seg}$. Image augmentation improves feature learning and enhances generalization. In addition, image augmentation is formed by incorporating various processes, like shifting, random erasing, and rotation^22^, which are epitomized below,

#### Random erasing

Random erasing is considered the augmentation approach that does not attempt to modify the pixel value of the single images in general. It is determined as the type of noise approach, which concentrates mostly on the local area instead of the single pixels, and the image attained using random erasing is symbolized as $\vartheta_{i}$.

#### Rotation

As a geometric method, rotation turns the image by a defined angle to the right or left, and the resulting image is referred to as $\vartheta_{j}$.

#### Shifting

The shifting is known as the method that includes the translation of an image inside its frame in vertical or horizontal directions for creating a new training sample. By accounting for changes in object position, it strengthens detection performance, and the resulting shifted image is denoted as $\vartheta_{o}$.

The augmented image is also generated by fusing the images obtained via shifting, rotation, and random erasing, and is indicated as,

7 $$\vartheta_{g} = \left\{ {\vartheta_{i} ,\vartheta_{j} ,\vartheta_{o} } \right\}$$

where, $\vartheta_{g}$ symbolizes the output attained using image augmentation.

### Feature extraction

The feature extraction process involves identifying and deriving meaningful attributes from source data for subsequent analysis and modeling tasks. It provides the dimensionality reduction of the data and improves detection accuracy by recognizing the most relevant information in the input. Here, the feature extraction is processed to mine features, such as Texture feature^25^, namely Maximal Correlation Coefficient^26^, Sum Entropy^28^, ASM^27^, Contrast^27^, WLBP^24^, and PHOG^23^. The image $\vartheta_{g}$ obtained by image augmentation is forwarded to feature extraction.

#### Texture feature

The process of determining the property or characteristic of the images that depict the spatial arrangement of patterns and pixel intensities inside the image is known as the texture feature^25^. The texture feature provided important data for tasks like segmentation, object detection, and classification of images. The texture features, considered the features namely Sum entropy, Maximal correlation coefficient, Contrast, and ASM, are extracted. Moreover, the texture feature is characterized as $f_{1}$.

- ASM

The process of measuring the uniform texture of the image is termed the ASM^27^, and it identifies the disorders present in the image texture. The highest value recorded by the ASM of 1. The maximum value of ASM is obtained when the grey-level distribution acquires a periodic constant form, and ASM is indicated as $A_{1}$.

8 $$A_{1} = \sum\limits_{x = 0}^{G - 1} {\sum\limits_{y = 0}^{G - 1} {\kappa \left( {x,y} \right)^{2} } }$$

where,*G* designates the gray level count, and $\kappa \left( {x,y} \right)$ indicates the normalized grayscale value at the location $\left( {x,y} \right)$ of the kernel with a total equivalent to 1.

- Contrast

The contrast^27^ is determined as the diverse moment of the Gray Level Co-Occurrence Matrix (GLCM), and it assesses the spatial frequency and the local variation present in the images. The contrast features also determine the variation among the minimal and maximal values of the adjacent set of pixels, and it is denoted as $A_{2}$,

9 $$A_{2} = \sum\limits_{x = 0}^{G - 1} {\sum\limits_{y = 0}^{G - 1} {\left( {x - y} \right)^{2} } }$$

- Sum entropy

The sum entropy^28^ is derived from the principle of entropy, which evaluates the sum of the randomness or uncertainty in a group of information or data. The sum entropy measures the variability and complexity present in the pixel values distribution, and it is indicated as $A_{3}$

10 $$A_{3} = - \sum {_{g} } \Psi_{h} \left( g \right)\log \Psi_{h} \left( g \right)$$

here, *g* indicating the intensity level, and $\Psi_{h}$ indicates the normalized sum distribution.

- Maximal correlation coefficient

The statistical approach evaluates the maximum degree of correlation among the regions in the segmented image, which is termed the maximal correlation coefficient^26^ and is used for obtaining the strongest non-linear or linear relationship. The maximal correlation coefficient is utilized for attaining the optimal conversions for the process of regression, and it is signified as $A_{4}$.

11 $$A_{4} = \sup \Upsilon \left( {u_{1} \left( P \right),u_{2} \left( Q \right)} \right)$$

where,*\sup* represents the supremum,$u_{1}$ and $u_{2}$ are Borell functions,*P* and *Q* indicates that the non-degenerates determine the value in the range $\left[ {0,1} \right]$.

The features like contrast, maximal correlation coefficient, ASM, and sum entropy, are unified to get the texture feature $f_{1}$ as expressed by,

12 $$f_{1} = \left\{ {A_{1} ,A_{2} ,A_{3} ,A_{4} } \right\}$$

#### PHoG

The PHoG^23^ is a spatial shape descriptor employed mostly in image classification. The spatial organization of edge patterns is obtained using PHoG and converted into a vector representation. Moreover, the Histogram of Orientation Gradients (HOG) is applied over every subregion at all resolutions in the image and is combined to get the PHOG feature. The PHOG extraction is detailed as follows. Initially, the edge contour extraction is performed. Then, the images are categorized into different cells at several pyramid levels. Afterwards, the HOG for all grids at all pyramid levels is measured. At last, the resultant PHOG descriptor for the images is represented as the combination of every HOG vector at all resolutions of the pyramids and is denoted as $f_{2}$.

#### WLBP

The WLBP^24^ is a texture descriptor utilized mostly in image analysis, especially for facial recognition and classification of the texture. WLBP efficiently incorporates the advantages of the Weber Local Descriptor (WLD) and the Local Binary Pattern (LBP). It encompasses two elements, such as LBP and differential excitation. The LBP and differential excitation at every pixel of the augmented image are measured, and they are combined to attain the WLBP feature. The WLBP method calculates the difference ratios between a target pixel and its surrounding pixels, represented as,

13 $$f_{3} = \sum\limits_{r = 0}^{I - 1} {H_{\zeta } } \left( {\frac{{\left| {c_{r} - c_{a} } \right|}}{{c_{a} }}} \right) \times 2^{r}$$

14 $$H_{\zeta } \left( c \right) = \left\{ {\begin{array}{*{20}c} {1,} & {c \ge \zeta } \\ {0,} & {c < \zeta } \\ \end{array} } \right.$$

where, *ζ* indicates the threshold for grey-level difference,$c_{a}$ is the center pixel, and $c_{r}$ its neighboring pixels, *I* indicates the neighboring pixel count, and $f_{3}$ is the WLBP feature.

By integrating WLBP, PHoG, and texture features, the feature vector *F* is constructed and expressed as,

15 $$F = \left\{ {f_{1} ,f_{2} ,f_{3} } \right\}$$

### Breast cancer detection

**Fig. 3:**
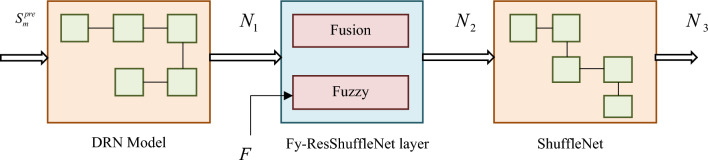
The general framework of Fuzzy RS-Net for the detection of breast cancer.

The Fuzzy RS-Net model is introduced for breast cancer detection using mammogram images. The Fuzzy RS-Net is established by formulating the Fuzzy concept^31^, DRN^29^, and ShuffleNet^30^. The Fuzzy RS-Net has three components, such as DRN, Fy-ResShuffleNet layer and ShuffleNet. First, the denoised image $S_{m}^{pre}$ is forwarded to the DRN model, and the resultant $N_{1}$ is attained. Then, the feature vector *F* as well as the outcome of the DRN $N_{1}$ is fed to the Fy-ResShuffleNet layer. In this layer, the fusion process is not performed as a simple weighted combination of feature vectors. Instead, fuzzy partitioning, membership function evaluation, and fuzzy rule generation are employed to model the uncertainty and imprecision associated with mammogram features. Here, fusion of the inputs is achieved by the application of the fuzzy concept, which is accomplished by employing the Taylor series^32^. The Taylor series formulation is utilized as a mathematical mechanism for adaptive feature fusion within the fuzzy framework. The generated fuzzy memberships and rules guide the fusion process by emphasizing discriminative mammographic characteristics while reducing the influence of uncertain feature responses. Furthermore, the Fy-ResShuffleNet outcome $N_{2}$ is fed into the ShuffleNet, and the output $N_{3}$ is obtained. Figure 3 depicts the general framework used for diagnosing breast cancer.

#### DRN model

The DRN model^29^ is a kind of Artificial Neural Network (ANN) architecture that uses residual learning by developing shortcut connections. The DRN utilizes shortcut connections that link input and output layers, thereby alleviating vanishing gradient issues in deeper networks and accelerating the training process. Moreover, the DRN is considered the fundamental architecture in DL. The DRN architecture consists of Batch Normalization (BN), a linear classifier, residual blocks, pooling layers, convolutional layers, and activation functions, which are described in detail below.

##### Convolutional layer

A key structure in DL networks, the convolutional layer, extracts feature from the image $S_{m}^{pre}$ automatically using a set of sufficiently deep kernels. The free parameters can be reduced by the 2D Convolutional Layer (Conv2d) during the training process, and the output of the Conv2d is expressed as,

16 $$Conv2d\left( k \right) = \sum\limits_{p = 0}^{q - 1} {\sum\limits_{s = 0}^{q - 1} {J_{p,s} * k_{{\left( {w + p} \right),\left( {z + s} \right)}} } }$$

In addition, the cross-correlation operator utilized in the Conv1d is formulated as,

17 $$Conv1d\left( k \right) = \sum\nolimits_{t = 0}^{{B_{wl - 1} }} {J_{t} * k}$$

here,* represents the cross-correlation operation, and *k* denotes the output of 2-D, the kernel matrix of dimensionality *q × q* is indicated as *J*,$J_{t}$ embodied the kernel size for the $t^{th}$ input neuron and *w* and *z* signifies the kernel position index.

##### Pooling layer

After the convolutional operation, this layer is applied to downsample the feature maps and improve model generalization by reducing overfitting. The process is formulated as,

18 $$C_{out} = \frac{{C_{wl} - t_{p} }}{L} + 1$$

19 $$s_{out} = \frac{{s_{wl} - t_{s} }}{L} + 1$$

where, the height and width of the input matrix are indicated as $s_{wl}$ and $C_{wl}$,$s_{out}$ and $C_{out}$ characterizes the width and height of the output,$t_{s}$ and $t_{p}$ stipulates the height and width of the kernel, and *L* is the stride.

##### Activation function

The activation function helps retain complex features, and ReLU, being straightforward, is generally more effective than the sigmoid function. The ReLU is represented as,

20 $$ReLU\left( k \right) = \left\{ {\begin{array}{*{20}c} {k,} & {k \ge 0} \\ {0,} & {k < 0} \\ \end{array} } \right.$$

where,*k* denotes the input features.

##### Batch normalization

BN helps minimize internal covariate shift by normalizing and scaling the activation function within the input layers. Furthermore, BN enhances the speed of the training approach and reduces the gradient explosion/vanishing and overfitting problems with the maximum training rate.

##### Residual block

A basic CNN incorporating a residual block transfers the input directly to the output using a shortcut connection. When there is a mismatch between input and output dimensions, a scaling factor is utilized to align the dimensions, as given in Eq. (20). If the dimensions of the input and output are the same, a direct shortcut connection is established between them, as shown in Eq. (21).

21 $$M = K\left( k \right) + C_{k} k$$

22 $$M = K\left( k \right) + k$$

where,$C_{k}$ embodies the matching factors size,*K* symbolizes the mapping relationship, and *M* and *k* indicates the output and input of the residual blocks.

##### Linear classifier

**Fig. 4:**
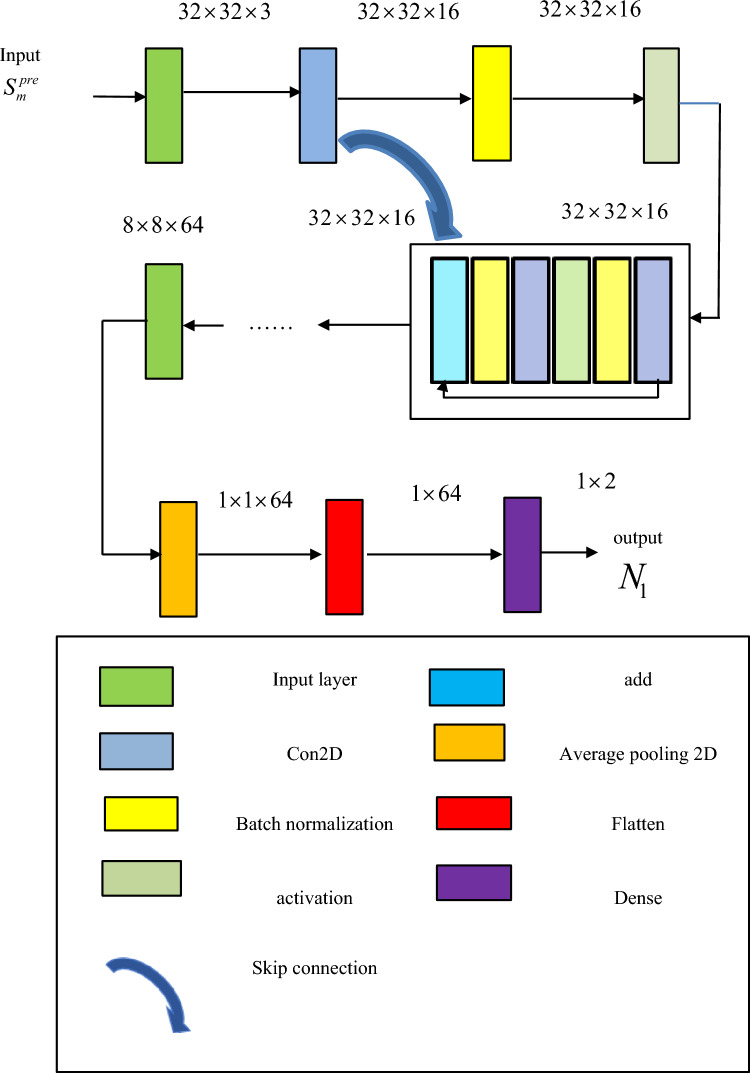
The framework for the DRN model.

The convolutional layer is responsible for extracting and compacting the input features, after which they are provided to the linear classifier to produce the final output. The network includes an FC layer and a softmax function, with the FC layer output determined using the dot product formulation presented in Eq. (22). The softmax is mostly exploited for the normalization of the input vectors. The value of the FC output is continuous, and it cannot represent the classification output, so a softmax function is used. The execution of the classification is expressed in Eq. (22),

23 $$N_{1} = C_{U \times V} * S_{m}^{pre} + X_{U \times V}$$

24 $$soft\max \left( {k_{w} } \right) = \frac{{e^{{k_{w} }} }}{{\sum\nolimits_{t = 1}^{{B_{Z} }} {e^{{k_{t} }} } }},w = 1,2,....B_{z}$$

where,$C_{U \times V}$ indicates the weight matrix of *U × V* size, $S_{m}^{pre}$ denotes the pre-processed image,$B_{z}$ embodies the resultant size, *X* specifies bias, and $k_{w}$ signifies the output layer element. The output for DRN is indicated as $N_{1}$. The framework for the DRN model is exhibited in Fig. 4.

The DRN model is incorporated in the proposed framework as a dedicated feature extraction module rather than being positioned as a shortcut branch within another network. Although DRN inherently employs residual connections through internal skip pathways, its role is to learn deep hierarchical representations from mammogram images and effectively address vanishing gradient issues. The DRN enables the extraction of discriminative and robust feature maps, which are subsequently processed by the ShuffleNet and fuzzy-based decision components. This design ensures that the advantages of residual learning are utilized for feature enhancement, while maintaining a structured pipeline for efficient classification and improved detection performance.

#### Fy-ResShuffleNet layer

The DRN model output $N_{1}$ as well as the feature vector *F* is passed to the Fy-ResShuffleNet layer. The fusion process is performed in the Fy-ResShuffleNet layer using the fuzzy concept^31^. The fuzzy logic is used to handle the imprecision and uncertainty in the data. Assume that a classification process in the 2D pattern $\left[ {0,1} \right]*\left[ {0,1} \right]$ as expressed as follows,

It is assumed that $u^{\prime}$ patterns are present, and are expressed as,

25 $$b_{W} = \left\{ {b_{W1} ,b_{W2} ,.....b_{{Wu^{\prime}}} } \right\}$$

The input patterns are designed regarding the features as follows,

26 $$F = \left\{ {f_{1} \left\| {f_{2} \left\| {f_{3} } \right.} \right.} \right\}$$

The process of fusion using the fuzzy approach in the Fy-ResShuffleNet layer is performed by considering the Taylor series^32^. From the Taylor series,

27 $$b\left( {x^{\prime} + 1} \right) = b\left( {x^{\prime}} \right) + \frac{{b^{\prime}\left( {x^{\prime}} \right)}}{1!}$$

28 $$b^{\prime}\left( {x^{\prime}} \right) = \frac{{b\left( {x^{\prime}} \right) - b\left( {x^{\prime} - \delta } \right)}}{\delta }$$

Substitute *δ = 1* and $b\left( {x^{\prime}} \right)$ in the Eq. (27)

29 $$b\left( {x^{\prime} + 1} \right) = b\left( {x^{\prime}} \right) + \frac{{b\left( {x^{\prime}} \right) - b\left( {x^{\prime} - 1} \right)}}{1!}$$

30 $$b\left( {x^{\prime} + 1} \right) = 2b\left( {x^{\prime}} \right) - b\left( {x^{\prime} - 1} \right)$$

Consider $b\left( {x^{\prime}} \right) = F$ and $b\left( {x^{\prime} - 1} \right) = N_{1}$, then the Eq. (30) becomes,

31 $$b_{W} = 2.F - N_{1}$$

1) Fuzzy partition

Let us contemplate that all axes of the pattern space are split into $\delta \left( {\delta \ge 2} \right)$ fuzzy subsets as $\left\{ {{D^\prime}_{1}^{\delta },\,{D^\prime}_{2}^{\delta },.....,{D^\prime}_{\delta}^{\delta} } \right\}$. The membership function expression is $\chi_{{a^{\prime}}}^{\delta } \left( b \right)$ for ${D^\prime}_{1}^{\delta }$ is expressed as,

32 $$\chi_{{a^{\prime}}}^{\delta } \left( b \right) = \max \left\{ {{{1 - \left| {b - \kappa_{{a^{\prime}}}^{\delta } } \right|} \mathord{\left/ {\vphantom {{1 - \left| {b - \kappa_{{a^{\prime}}}^{\delta } } \right|} {\upsilon^{\delta } }}} \right. \kern-0pt} {\upsilon^{\delta } }},0} \right\}a^{\prime} = 1,2,.....,\delta \left( {\delta \ge 2} \right)$$

33 $$\kappa_{{a^{\prime}}}^{\delta } = {{\left( {a^{\prime} - 1} \right)} \mathord{\left/ {\vphantom {{\left( {a^{\prime} - 1} \right)} {\left( {\delta - 1} \right)}}} \right. \kern-0pt} {\left( {\delta - 1} \right)}},a^{\prime} = 1,2,.....,\delta$$

34 $$\upsilon_{{}}^{\delta } = {1 \mathord{\left/ {\vphantom {1 {\left( {\delta - 1} \right)}}} \right. \kern-0pt} {\left( {\delta - 1} \right)}}$$

For *δ = 1*, the membership function ${D^\prime}_{{a^{\prime}}}^{1}$ is arbitrated as,

35 $$\chi_{{a^{\prime}}}^{1} \left( b \right) = \left\{ {\begin{array}{*{20}c} {1,} & {if0 \le b \le 1\,} \\ {0,} & {otherwise} \\ \end{array} } \right.$$

where, the fuzzy subset ${D^\prime}_{{a^{\prime}}}^{1}$ is in the range $\left[ {0,1} \right]$. The mammogram features obtained during extraction are mapped to three linguistic categories: Low, Medium, and High. The membership functions are constructed using the statistical distribution of texture, PHOG, and WLBP features obtained from mammogram images. The pattern space is divided according to the membership functions to construct various fuzzy partitions.

2) Rule generation

The rules for every subset are generated after the partition is created. The fuzzy rule $O_{{a^{\prime},\beta }}^{{c^{\prime},\alpha }}$ present in the 2D classification system is modelled as,

Rule $O_{{a^{\prime},\beta }}^{{d^{\prime},\alpha }}$: If $b_{W2}$ is ${D^\prime}_{\beta }^{{d^{\prime}}}$ and $b_{W1}$ is ${D^\prime}_{{a^{\prime}}}^{{c^{\prime}}}$ then $\left( {b_{W1} ,b_{W2} } \right)$ is associated with the class $T_{{a^{\prime},\beta }}^{{d^{\prime},\alpha }}$ with the ${Tc^\prime} = {Tc^\prime}_{{{a^\prime},\beta }}^{{{d^\prime},\alpha }}$,

36 $$a^{\prime} = 1,2,.....,d^{\prime};d^{\prime} = 1,2,.......,d^{\prime}_{\max }$$

37 $$\beta = 1,2,.....,\alpha ;\alpha = 1,2,.......,\alpha_{\max }$$

The consequent $T_{{a^{\prime},\beta }}^{{d^{\prime},\alpha }}$ and certainty ${Tc^\prime}_{{a^{\prime},\beta }}^{{d^{\prime},\alpha }}$ of each rule can be determined by using the ensuing process. Further, $T_{{a^{\prime},\beta }}^{{d^{\prime},\alpha }}$ represent one of the *Γ* classes and the maximal count of fuzzy subspaces is signified as $d^{\prime}_{\max } \times \alpha_{\max }$.

Method 1): Fuzzy rule generation

The fuzzy rule generation process is adapted for breast cancer detection by employing mammogram-specific descriptors, namely Texture, PHOG, and WLBP features. These descriptors characterize tissue heterogeneity, lesion boundary structures, and local texture variations present in mammogram images. The fuzzy partitions and membership functions are therefore constructed from mammogram-derived feature distributions, enabling the generated rules to model uncertainty associated with benign and malignant breast lesions. In the proposed breast cancer detection framework, the fuzzy rules are generated using mammogram-specific descriptors, namely texture, PHOG, and WLBP features.

- Rule 1: IF Texture is High AND PHOG is High THEN Malignant.
- Rule 2: IF Texture is Low AND WLBP is Low THEN Benign.
- Rule 3: IF Texture is Medium AND PHOG is High THEN Suspicious/Malignant.

These rules enable the system to handle uncertainty in mammogram characteristics and improve classification robustness.

a) Evaluate $Z_{T\mu }$ for $\mu = 1,2,....,\Gamma$ as,

38 $$Z_{T\mu } = \sum\limits_{{b^{\prime}_{W} }} {\chi_{{a^{\prime}}}^{{d^{\prime}}} } \left( {b_{W1} } \right) * \chi_{\beta }^{\alpha } \left( {b_{W2} } \right)$$

b) Identify class $\raisebox{3pt}{-}\kern-5pt{\lambda} \left( {T\raisebox{3pt}{-}\kern-5pt{\lambda} } \right)$ in such a way,

39 $$Z_{T\raisebox{3pt}{-}\kern-5pt{\lambda}} = \max \left\{ {Z_{T1} ,Z_{T2} ,.....Z_{{Tn^{\prime}}} } \right\}$$

$T_{{a^{\prime},\beta }}^{{d^{\prime},\alpha }}$ is detected as a class $\raisebox{3pt}{-}\kern-5pt{\lambda} \left( {T\raisebox{3pt}{-}\kern-5pt{\lambda} } \right)$ in $Z_{T\raisebox{3pt}{-}\kern-5pt{\lambda} }$, when a class acquires the highest value.

c) When a class acquires the maximum value in the $Z_{T\raisebox{3pt}{-}\kern-5pt{\lambda} }$ and the $T_{{a^{\prime},\beta }}^{{d^{\prime},\alpha }}$ is expressed as,

40 $$T_{{a^{\prime},\beta }}^{{d^{\prime},\alpha }} = {{\left( {Z_{T\raisebox{3pt}{-}\kern-5pt{\lambda} } - Z} \right)} \mathord{\left/ {\vphantom {{\left( {Z_{T\raisebox{3pt}{-}\kern-5pt{\lambda} } - Z} \right)} {\sum\limits_{\mu = 1}^{\Gamma } {Z_{T\mu } } }}} \right. \kern-0pt} {\sum\limits_{\mu = 1}^{\Gamma } {Z_{T\mu } } }}$$

here,

41 $$Z = \mathop {{{\sum\limits_{\mu = 1}^{\Gamma } {Z_{T\mu } } } \mathord{\left/ {\vphantom {{\sum\limits_{\mu = 1}^{\Gamma } {Z_{T\mu } } } {\left( {\Gamma - 1} \right)}}} \right. \kern-0pt} {\left( {\Gamma - 1} \right)}}}\limits_{T\mu \ne T\raisebox{3pt}{-}\kern-5pt{\lambda} }$$

In this course, the consequent $T_{{a^{\prime},\beta }}^{{d^{\prime},\alpha }}$ is articulated as the class $\raisebox{3pt}{-}\kern-5pt{\lambda} \left( {T\raisebox{3pt}{-}\kern-5pt{\lambda} } \right)$, which has a maximum total of $\chi_{{a^{\prime}}}^{{d^{\prime}}} \left( {b_{W1} } \right) \times \chi_{\beta }^{\alpha } \left( {b_{{W_{2} }} } \right)$ among the *Γ* classes as shown in Eq. (38). Besides, a dummy rule is generated at the fuzzy subspace ${D^\prime}_{{a^{\prime}}}^{{d^{\prime}}} \times {D^\prime}_{\beta }^{\alpha }$, when there is no pattern in the subspace.

Assume a set of $\left( {1 + 2 + .... + d^{\prime}_{\max } } \right) \times \left( {1 + 2 + .... + \alpha_{\max } } \right)$ fuzzy rules are produced by the $\varphi_{all}$:

42 $$\varphi_{all} = \left\{ {O_{{a^{\prime},\beta }}^{{d^{\prime},\alpha }} :a^{\prime} = 1,2,.....,d^{\prime};d^{\prime} = 1,2,........,d^{\prime}_{\max } } \right\},\left\{ {\beta = 1,2,.......,\alpha ;\alpha = 1,2,........,\alpha_{\max } } \right\}$$

43 $$\varphi^{{_{{d^{\prime},\alpha }} }} = \left\{ {O_{{a^{\prime},\beta }}^{{d^{\prime},\alpha }} :a^{\prime} = 1,2,.....,d^{\prime};\beta = 1,2,.......,\alpha } \right\},\left\{ {d^{\prime} = 1,2,........,d^{\prime}_{\max } ;\alpha = 1,2,........,\alpha_{\max } } \right\}$$

Further, a group of fuzzy rules $\varphi_{{d^{\prime},\alpha }}$ in the fuzzy partition produced by the $d^{\prime} \times \alpha$ fuzzy grid. The rule set $\varphi_{all}$ is signified as,

44 $$\varphi_{all} = \mathop \cup \limits_{{d^{\prime} = 1}}^{{d^{\prime}_{\max } }} \mathop \cup \limits_{\alpha = 1}^{{\alpha_{\max } }} \varphi^{{_{{d^{\prime},\alpha }} }}$$

The main purpose of the rule selection issue is to remove every redundant fuzzy rule and select the important fuzzy rules by utilizing $\varphi_{all}$.

3) Classification of a new pattern

Assumes a fuzzy classification model is produced by using the subset *φ* of the rule set $\varphi_{all}$. The new pattern $b_{W} = \left\{ {b_{W1} ,b_{W2} } \right\}$ is categorized using the fuzzy rules in *φ* as expressed using the below-represented method,

Method 2) New pattern classification

a) Evaluate pattern $\wp_{T\mu }$ for class $\mu = 1,2,.....,\Gamma$ as,

45 $$\wp_{T\mu } = \max \left\{ {\chi_{{a^{\prime}}}^{{d^{\prime}}} \left( {b_{W1} } \right) \times \chi_{\beta }^{\alpha } \left( {b_{W2} } \right) \times {Tc^\prime}_{{a^{\prime},\beta }}^{{d^{\prime},\alpha }} .T_{{a^{\prime},\beta }}^{{d^{\prime},\alpha }} = class\mu \,\,and\,O_{{a^{\prime},\beta }}^{{d^{\prime},\alpha }} \in \varphi } \right\}$$

b) Discover the class $\raisebox{3pt}{-}\kern-5pt{\lambda} \left( {T\raisebox{3pt}{-}\kern-5pt{\lambda} } \right)$ so that

46 $$\wp_{T\mu } = N_{2} = \max \left\{ {\wp_{T1} ,\wp_{T2} ,....,\wp_{T\Gamma } } \right\}$$

When multiple classes record the maximum value in the $\raisebox{3pt}{-}\kern-5pt{\lambda} \left( {T\raisebox{3pt}{-}\kern-5pt{\lambda} } \right)$, then the classification of the $b_{W}$ is rejected, or $b_{W}$ is assigned to the class $\raisebox{3pt}{-}\kern-5pt{\lambda} \left( {T\raisebox{3pt}{-}\kern-5pt{\lambda} } \right)$ indicated as $\wp_{T\mu }$. The term $N_{2}$ represents the output for the Fy-ResShuffleNet layer.

#### ShuffleNet model

ShuffleNet^30^ is a kind of CNN that is developed for effective image classification, especially in embedded and mobile devices. The output $N_{2}$ of the Fy-ResShuffleNet layer is given as input to the ShuffleNet. The ShuffleNet applied novel architectural techniques known as channel shuffle to enhance the flow of the data among the various layers while ensuring a lightweight design. Although ShuffleNet is originally designed to operate on raw or intermediate feature maps, in the proposed model, it is intentionally applied after feature fusion to refine and compress the combined representation. The fused vector already contains complementary information from deep residual features, handcrafted descriptors, and fuzzy-weighted interactions, which collectively provide a rich high-level semantic embedding. In this context, ShuffleNet is not used as a primary feature extractor but as a lightweight feature refinement and re-encoding module. Its channel shuffle and group convolution operations help to reorganize the fused feature space, reduce redundancy, and improve discriminative capability while maintaining computational efficiency. Therefore, instead of processing raw image data, ShuffleNet operates on an enriched feature representation to enhance compactness and generalization performance of the final classification stage. The ShuffleNet is considered a multilayer architecture comprised of Pooling, ReLU, Convolutional layer, SoftMax, BN, and FC layer. Furthermore, pooling layers are incorporated in ShuffleNet to minimize the computational cost.

As the first layer of the architecture, the input layer provides data to the convolutional layers, which apply kernels to obtain feature representations. The convolutional layer output can be expressed as,

47 $$M^{\prime}\left( {v^{\prime},t^{\prime}} \right) = \left( {E^{\prime} \times R^{\prime}} \right)\left( {v^{\prime},t^{\prime}} \right) = \sum\limits_{{g^{\prime}}} {\sum\limits_{{h^{\prime}}} {E^{\prime}\left( {h^{\prime},g^{\prime}} \right)} } R^{\prime}\left( {v^{\prime} - h^{\prime},t^{\prime} - g^{\prime}} \right)$$

where, $M^{\prime}$ depicts the resultant feature map,$E^{\prime}$ indicates the input, and $R^{\prime}$ symbolizes the convolutional kernel. After the operation of the convolutional layer on the input, the resultant dimension $r^{\prime} = \left( {{{\left( {v^{\prime} - w^{\prime} + 2z^{\prime}} \right)} \mathord{\left/ {\vphantom {{\left( {v^{\prime} - w^{\prime} + 2z^{\prime}} \right)} {\left( {\eta + 1} \right)}}} \right. \kern-0pt} {\left( {\eta + 1} \right)}}} \right)$ is attained. Here, the $z^{\prime}$ indicates the padding, *η* signifies stride and $w^{\prime}$ signifies the dimension of the kernel.

The three types of convolutional operations, namely two pointwise group convolutions and a depthwise convolution, are combined to develop the ShuffleNet unit. In this case, BN follows the initial pointwise group convolution, and thereafter, ReLU and channel shuffle operations are performed. The most effective and straightforward activation is the ReLU activation and is represented as

48 $$q^{\prime}\left( {r^{\prime}} \right) = \left\{ {\begin{array}{*{20}c} {0,} & {r^{\prime} < 0} \\ {r^{\prime}} & {r^{\prime} \le 0} \\ \end{array} } \right.$$

**Fig. 5:**
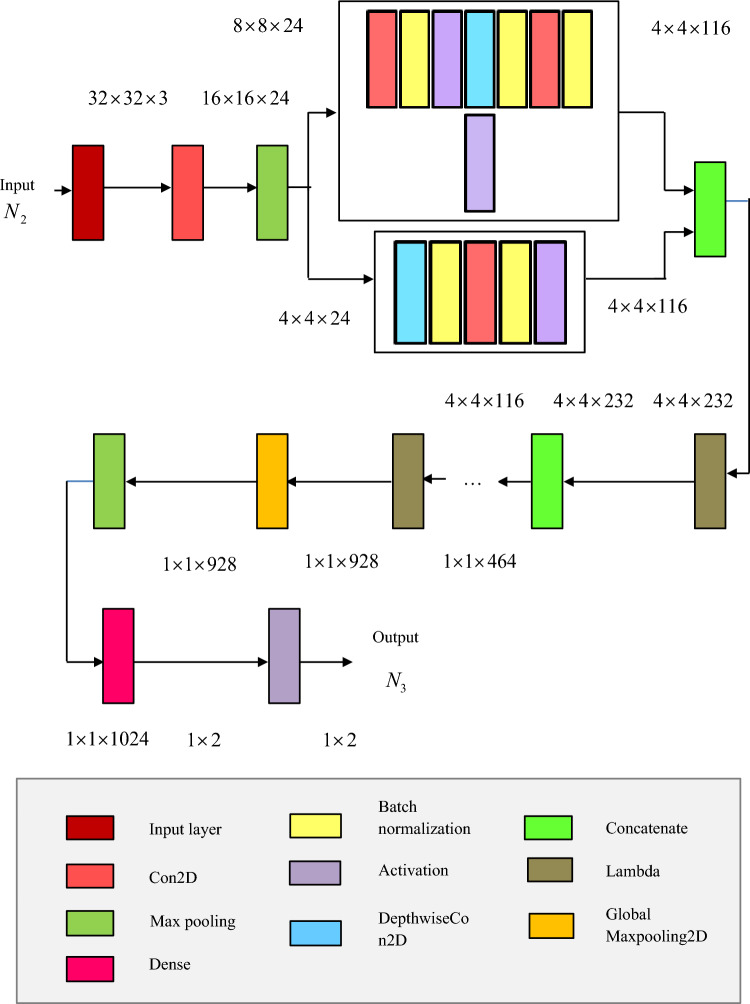
The schematical representation of the ShuffleNet model.

Furthermore, ReLU is employed to deactivate neurons when the input is negative and activate them when it is positive. The second and third convolutions utilize point-wise and depth-wise convolutions, respectively, after which BN is applied. Consecutive ShuffleNet units extract features, and the resulting output is fed into the FC and SoftMax layers for classification. The mathematical expression for the FC layer output is given as,

49 $$N_{3} = \sum\limits_{{t^{\prime} = 0}}^{{h^{\prime} \times g^{\prime}}} {K^{\prime}_{{v^{\prime}t^{\prime}}} } \times {N_2}_{{v^{\prime}}} + {m^\prime}_{{v^{\prime}}}$$

here, $g^{\prime},h^{\prime}$ and $v^{\prime}$ indicates the height, width, and index for the output in the FC layer, $m^{\prime}$ and $K^{\prime}$ specifies the bias and weight, and the final output for the ShuffleNet is depicted as $N_{3}$, which signifies the breast cancer detected output. The structural overview of the ShuffleNet model is depicted in Fig. 5.

## Results and discussion

The following section discusses the comparative performance analysis and experimental findings of the Fuzzy RS-Net approach for breast cancer detection, covering the dataset, experimental environment, and evaluation criteria.

### Experimental setup

The proposed Fuzzy RS-Net method is developed in Python and utilized for breast cancer detection. To ensure reliable and fair evaluation, all baseline models and the proposed Fuzzy RS-Net are trained and tested using identical experimental settings with multiple data split configurations. Table 1 presents the random erasing settings. Tables 2 and 3 depict the layer details of DRN and ShuffleNet and the parameters of the Fuzzy RS-Net approach.

**Table 1:** Random erasing parameters.

Parameters	Values
Erasing probability (p)	0.5
Area ratio (s)	0.02 to 0.4
Aspect ratio (r)	0.03

**Table 2:** DRN and ShuffleNet layer details.

Model	Layer name	No. of layers	Neurons
***DRN***	Input layer	1	-
	Convolution layer	1	64
	Pooling layer	1	-
	Residual block	2	64, 128
	DRN block	3	256, 512, 512
	Fully connected	1	256
	Dropout layer	1	-
	Output layer	1	-
***ShuffleNet***	Input layer	1	-
	Convolution layer	1	24
	Pooling layer	1	-
	Shuffle unit	4	240, 480, 960, 1024
	Dense layer	1	512
	Dropout layer	1	-
	Output layer	1	-

**Table 3:** Parameters of Fuzzy RS-Net.

Parameter	Value
Epoch	120
Batch size	16
Optimizer	Adam
Kernel size	(3,3)
Dropout	0.2
Beta 1	0.99
Beta 2	0.999
Epsilon	0.0001
Learning rate	0.01
Loss function	Binary cross entropy
Activation	Sigmoid

### Evaluation measures

The valuation parameters, namely sensitivity, specificity, accuracy, and Receiver Operating Characteristics (ROC), are utilized for executing the analysis of Fuzzy RS-Net.

#### Accuracy

The accuracy can be demarcated as the ratio of evaluating the correctness of the Fuzzy RS-Net correlated to the actual result, and it is indicated as *ℑ*,

50 $$\Im = \frac{{{\rm M}{\rm O} + {\rm M}{\rm A}}}{{{\rm M}{\rm O} + {\rm M}{\rm A} + {\rm I}{\rm O} + {\rm I}{\rm A}}}$$

wherein,${\rm I}{\rm O}$ symbolizes false positive,${\rm M}{\rm A}$ implies true negative, ${\rm M}{\rm O}$ designates true positive, and ${\rm I}{\rm A}$ characterizes false negatives.

#### Specificity

Specificity measures the percentage of healthy samples correctly classified by Fuzzy RS-Net and is expressed as *Ξ*,

51 $$\Xi = \frac{{{\rm M}{\rm A}}}{{{\rm M}{\rm A} + {\rm I}{\rm O}}}$$

#### Sensitivity

The sensitivity is the percentage of breast cancer samples detected precisely by Fuzzy RS-Net and is represented as *ℜ*.

52 $$\Re = \frac{{{\rm M}{\rm O}}}{{{\rm M}{\rm O} + {\rm I}{\rm A}}}$$

#### Receiver operating characteristics (ROC)

To analyze the model’s performance under different threshold values, the ROC curve is generated by displaying the relationship between the True Positive Rate (TPR) and False Positive Rate (FPR).

### Dataset description

The mammogram images used for detecting breast cancer in this work are taken from the Digital Database for Screening Mammography (DDSM), the Mammographic Image Analysis Society (MIAS) datasets^33^, and the Curated Breast Imaging Subset of DDSM (CBIS-DDSM)^34^^35^.

#### DDSM dataset

The DDSM dataset includes a collection of 2500 mammogram studies that are used for breast cancer detection and analysis. Two types of breast images, together with aggregated patient data, are included in the dataset, with suspicious regions labeled at the pixel level to identify the position and type of abnormalities. The processed dataset includes 2620 mammogram images, with 1609 normal and 1011 abnormal cases, and a high spatial resolution of *2866 × 5414* pixels at 96 dpi. These characteristics make the dataset suitable for accurate training and evaluation by providing a detailed representation of both normal and suspicious breast tissue patterns.

#### MIAS dataset

The MIAS dataset includes 322 digitized mammograms with 270 normal and 52 abnormal cases. These films were originally on a 2.3 GB 8 mm tape and were subsequently processed and reduced to a resolution of 200 microns per pixel. The resulting digital mammograms are available in a standardized spatial resolution of *1024 × 1024* pixels at 96 dpi, enabling consistent analysis for breast cancer detection.

#### CBIS-DDSM dataset

The CBIS-DDSM dataset serves as a public mammography resource for breast cancer detection and research applications. It provides annotated, high-resolution digitized mammogram images to assist in the creation and evaluation of computer-aided diagnosis frameworks. The dataset contains a total of 1696 mammogram images, which include both normal and abnormal cases. Specifically, it consists of 914 normal images and 786 abnormal images representing different breast conditions. All images in this dataset are high-resolution mammograms with a resolution of *4126 × 5491* pixels at 96 dpi, which allows detailed analysis of breast tissue structures. This dataset is widely used in DL-based medical image analysis because of its reliability, structured annotations, and suitability for training and testing classification models for breast cancer detection.

The impact of image augmentation is explicitly clarified in terms of dataset size. For the DDSM dataset, 2620 images are available before augmentation, increasing to 7860 after applying augmentation techniques such as rotation, shifting, and random erasing, thereby improving model generalization and mitigating overfitting. For the MIAS dataset, 322 images are present before augmentation, and this number increases to 966 images after augmentation. In the CBIS-DDSM dataset, image augmentation increases the total number of samples from 1696 to 5088, which improves dataset variability and contributes to better robustness and generalization performance of the proposed approach.

Class imbalance during training is addressed using data augmentation techniques. As the datasets may contain unequal numbers of normal and abnormal cases, data augmentation methods, such as rotation, translation, and random erasing, are applied to increase the representation of minority class samples. This helps achieve a more balanced dataset, reduces classification bias, and enhances the model’s capability to extract meaningful features from both classes. The augmentation process is carried out only on the training data after dividing the dataset into training, validation, and testing sets at the image level. Following this split, augmentation methods including rotation, shifting, and random erasing are applied to the training subset to increase sample diversity and improve class distribution when normal and abnormal cases are unevenly represented. The validation and testing sets are kept unchanged throughout the process to ensure unbiased evaluation of the model performance. Since augmentation is restricted exclusively to the training set, all transformed samples remain within the training data, preventing any data leakage or overlap across splits. This ensures dataset independence, maintains evaluation integrity, and provides a fair assessment of the model’s generalization capability.

For dependable evaluation of the model, the dataset is split into training, testing, and validation sets using different experimental arrangements. In the 90%–5%–5% partition, the DDSM dataset includes 7074 training, 393 testing, and 393 validation images, the MIAS dataset includes 870 training, 48 testing, and 48 validation images, and the CBIS-DDSM dataset includes 4579 training, 255 testing, and 254 validation images, and the same split configuration is consistently applied to both the proposed method and all baseline models. In the 80%–10%–10% split, the DDSM dataset consists of 6288 training, 786 testing, and 786 validation images; the MIAS dataset consists of 773 training, 97 testing, and 96 validation images; and the CBIS-DDSM dataset consists of 4070 training, 509 testing, and 509 validation images. In the 70%–15%–15% split, the DDSM dataset includes 5502 training, 1179 testing, and 1179 validation images; the MIAS dataset includes 676 training, 145 testing, and 145 validation images; and the CBIS-DDSM dataset includes 3562 training, 763 testing, and 763 validation images. Under the 60%–20%–20% data split, the DDSM dataset contains 4716 training images, 1572 testing images, and 1572 validation images. Similarly, the MIAS dataset includes 580 training, 193 testing, and 193 validation images, while the CBIS-DDSM dataset comprises 3053 training, 1018 testing, and 1018 validation images. The proposed model and all existing benchmark methods are tested using consistent dataset partitions from the DDSM, MIAS, and CBIS-DDSM datasets, ensuring an objective performance evaluation. By maintaining identical training, testing, and validation splits for all methods, the evaluation remains consistent and fair across all experiments. The preprocessing steps are applied in a consistent manner across all images in all three datasets to ensure uniformity in input data. Wavelet domain filtering is performed on every mammogram image to reduce noise and enhance relevant features before further processing. This consistent preprocessing strategy ensures fair comparison and reliable feature extraction throughout the training and testing phases.

### Image outcome

The images obtained during the analysis of abnormal or normal images from the DDSM and MIAS datasets are exhibited below. The regions of an image that influence the model’s predictions are visually identified using heatmaps. It maps the importance of different areas using intensity variations, enabling clear identification of relevant features. In medical image analysis, heatmaps help in localizing suspicious or abnormal regions effectively. By showing which regions influence the model’s predictions, heatmaps improve interpretability, increase transparency, and foster confidence in the model, especially for clinical applications.

#### DDSM dataset

The sample images obtained when the DDSM dataset is applied to the Fuzzy RS-Net are shown in this segment.

i) With abnormal

**Fig. 6:**
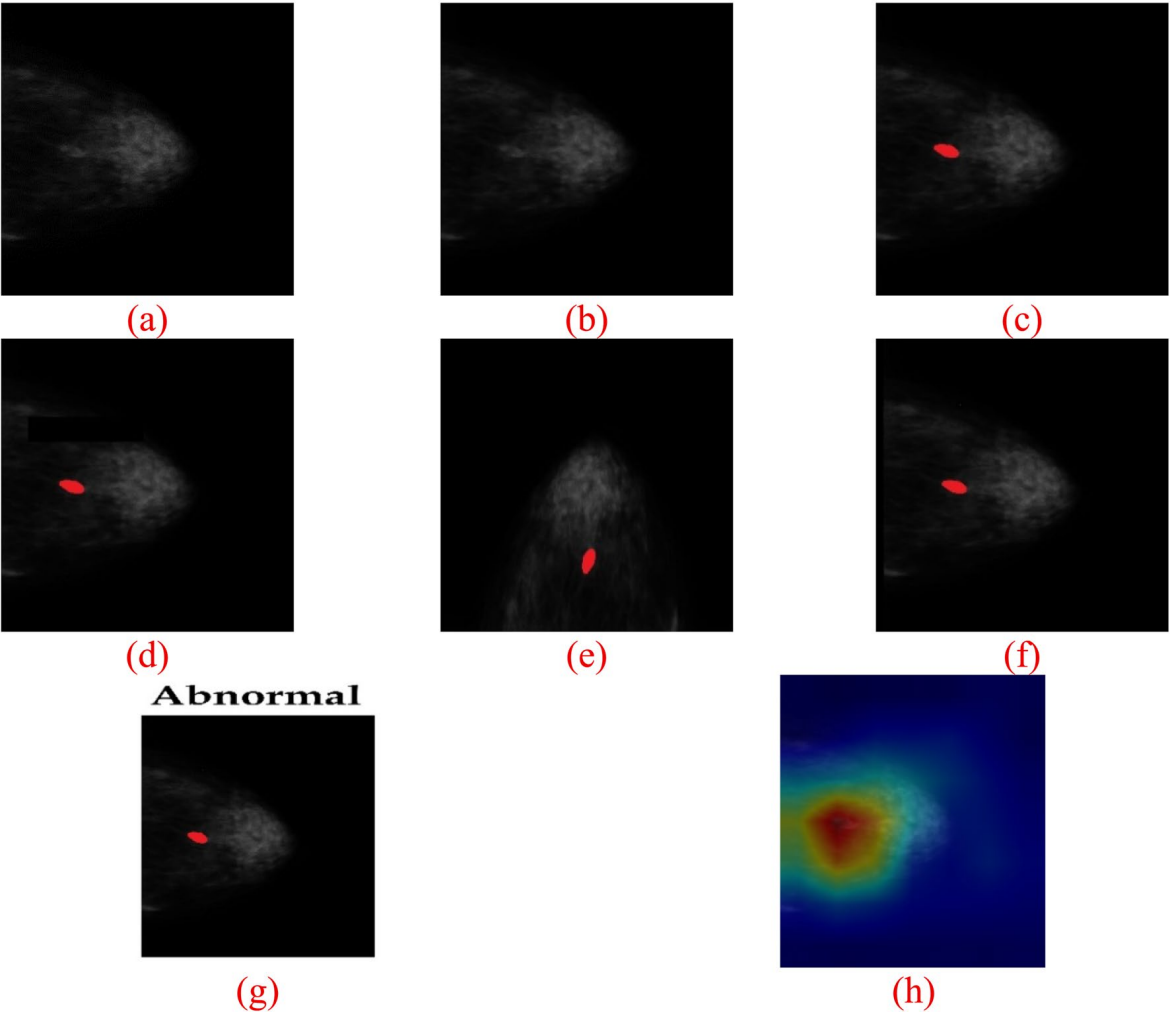
Image outcome for the Fuzzy RS-Net using the abnormal images from the DDSM dataset (**a**) input, (**b**) pre-processed, (**c**) segmented, (**d**) random-erased, (**e**) rotation-augmented, (**f**) shifting-augmented images, (**g**) detected output, and (**h**) heatmap.

The image result for the presented Fuzzy RS-Net in view of the DDSM dataset when applied with the abnormal image is epitomized in Fig. 6. The input and pre-processed images are portrayed in Figs. 6 (a) and 6(b). Figures 6 (c) and 6(d) signify the segmented and random-erased images. The rotation, and shifting-augmented images, and detected output are characterized in Figs. 6 (e),6(f) and 6(g). In Fig. 6 (h), the heatmap for the detected output is presented.

ii) With normal

**Fig. 7:**
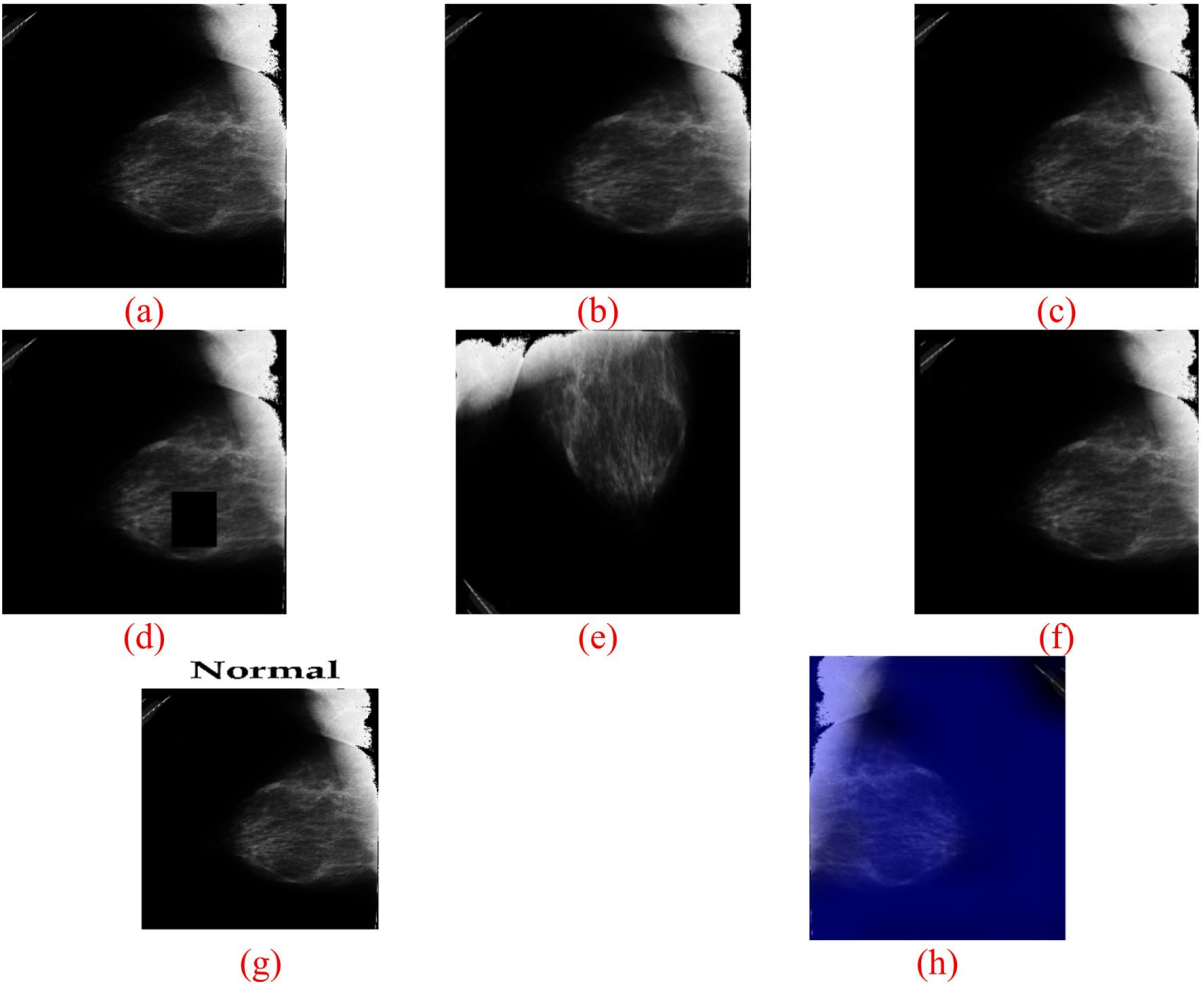
Image result for the Fuzzy RS-Net for a normal image from the DDSM dataset (**a**) input, (**b**) pre-processed, (**c**) segmented, (**d**) random-erased, (**e**) rotation-augmented, (**f**) shifting-augmented images, (**g**) detected output, and (**h**) heatmap.

Figure 7 depicts the result generated by the Fuzzy RS-Net for normal images from the DDSM dataset. Figure 7 (a) and 7(b) epitomizes the input and pre-processed images. Figures 7 (c) and 7(d) depict the segmented and random-erased images. Figures 7 (e), 7(f), and 7(g) portray the rotation, the shifting-augmented images, and the detected output images. The heatmap for the output image is demonstrated in Fig. 7 (h).

#### MIAS dataset

In this section, the sample images resulting from feeding MIAS dataset images into the Fuzzy RS-Net are demonstrated.

i) With abnormal

**Fig. 8:**
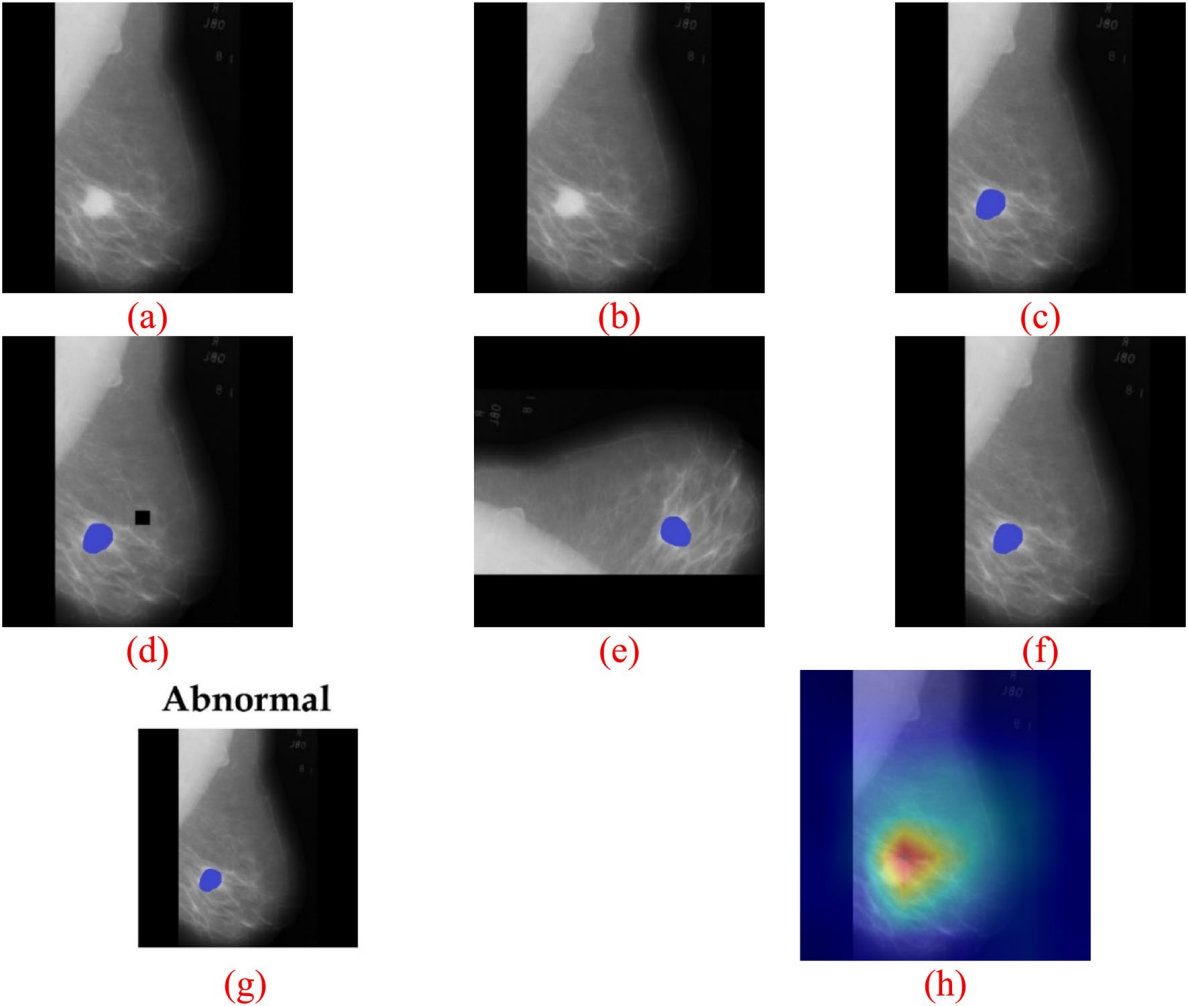
Image output for the Fuzzy RS-Net regarding the abnormal image from the MIAS dataset (**a**) input, (**b**) pre-processed, (**c**) segmented, (**d**) random-erased, (**e**) rotation-augmented, (**f**) shifting-augmented images, (**g**) detected output, and (**h**) heatmap.

The resultant images recorded by the Fuzzy RS-Net based on the abnormal image from the MIAS dataset are presented in Fig. 8. The input and pre-processed images are symbolized in Fig. 8 (a) and Fig. 8(b). The segmented and random-erased images are specified in Fig. 8(c) and Fig. 8(d). The rotation-augmented, shifting-augmented, and detected output images are figured in Figs. 8 (e), 8(f), and 8(g). Figure 8(h) depicts the heatmap for the output image.

ii) With normal

**Fig. 9:**
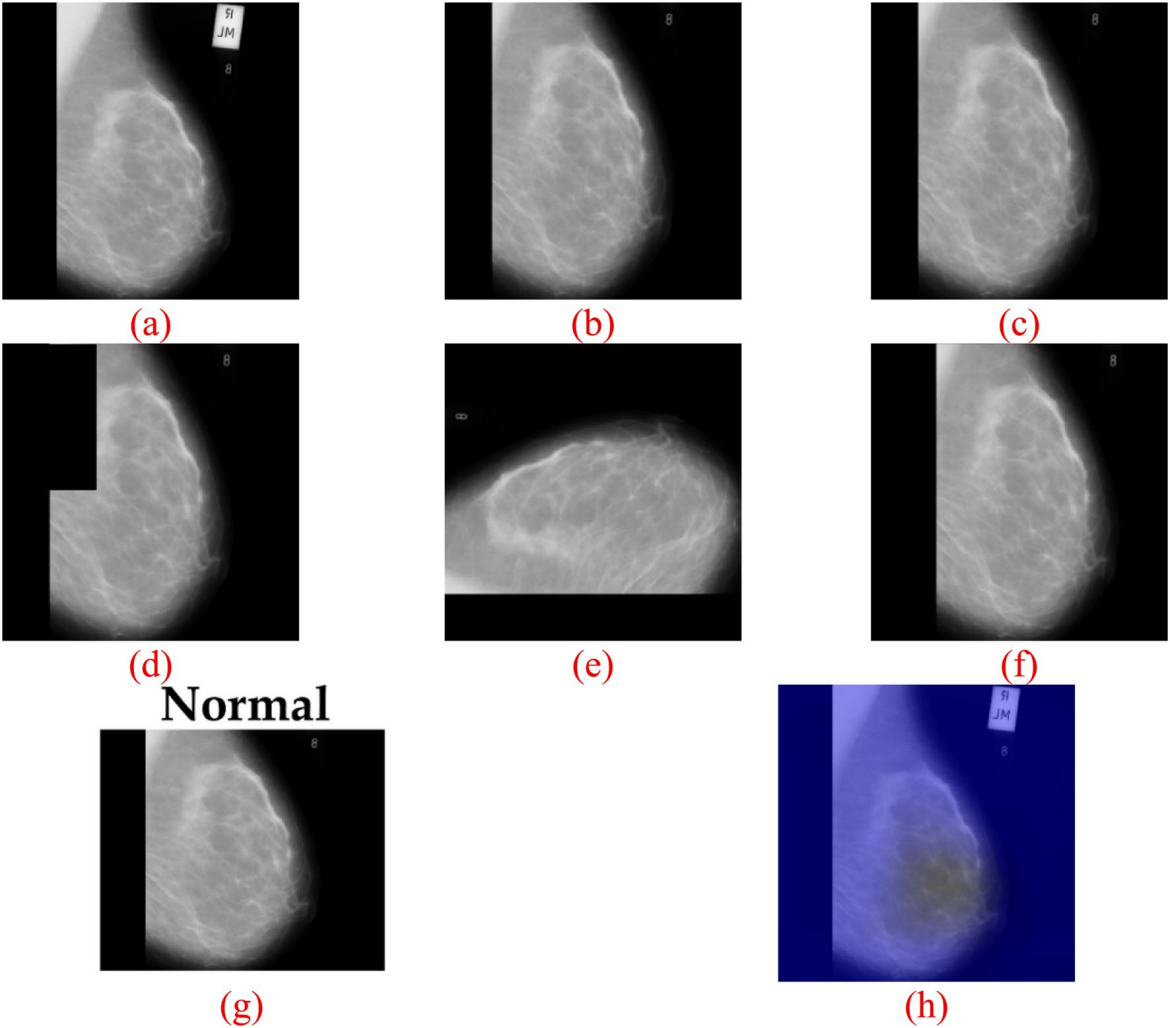
Image resultant for the Fuzzy RS-Net for the MIAS dataset (**a**) input, (**b**) pre-processed, (**c**) segmented, (**d**) random-erased, (**e**) rotation-augmented, (**f**) shifting-augmented images, (**g**) detected output, and (**h**) heatmap.

The images generated by the Fuzzy RS-Net for the normal sample in the MIAS dataset are illustrated in Fig. 9. The input and pre-processed images are revealed in Fig. 9 (a) and Fig. 9(b). Figure 9 (c) and Fig. 9(d) exhibit the segmented and random-erased images. The rotation-augmented, shifting-augmented, and detected output images are explicated in Figs. 9 (e), 9(f), and 9(g). The heatmap for the detected output is illustrated in Fig. 9(h).

#### CBIS-DDSM dataset

This section presents sample results generated by applying the Fuzzy RS-Net to images from the CBIS-DDSM dataset.

i) With abnormal

**Fig. 10:**
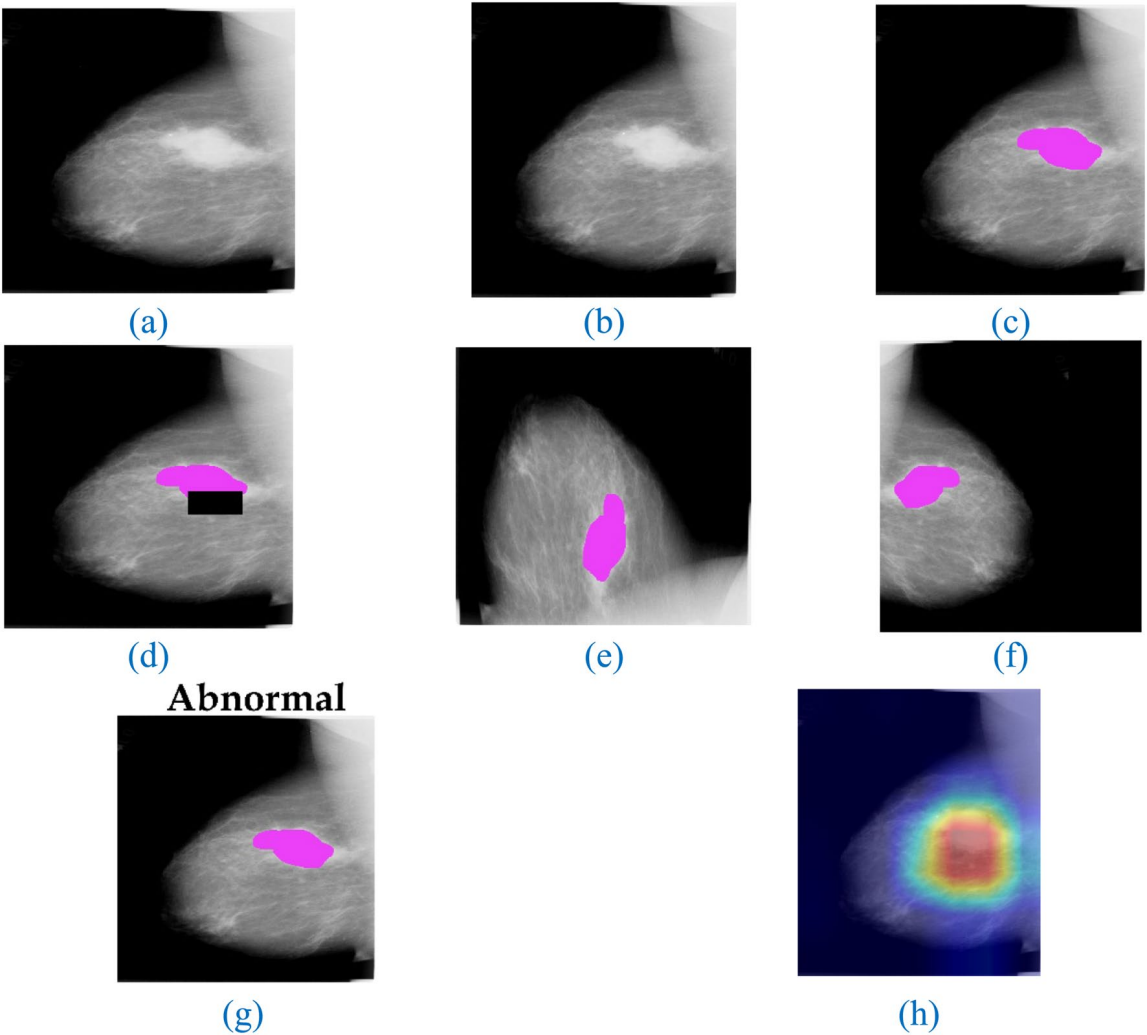
The resultant image generated by the Fuzzy RS-Net based on an abnormal CBIS-DDSM dataset image (a) input, (b) pre-processed, (c) segmented, (d) random-erased, (e) rotation-augmented, (f) shifting-augmented images, g) detected output, and h) heatmap.

Figure 10 presents the resultant images obtained from the Fuzzy RS-Net model using an abnormal CBIS-DDSM sample. The input image and its pre-processed version are indicated in Fig. 10(a) and Fig. 10(b). The segmentation result and random-erased image are displayed in Fig. 10(c) and Fig. 10(d). Figure 10(e), Fig. 10(f) and Fig. 10(g) show the rotation-augmented image, shifting-augmented image, and detected output image, respectively, whereas Fig. 10(h) illustrates the heatmap of the final output.

ii) With normal

**Fig. 11:**
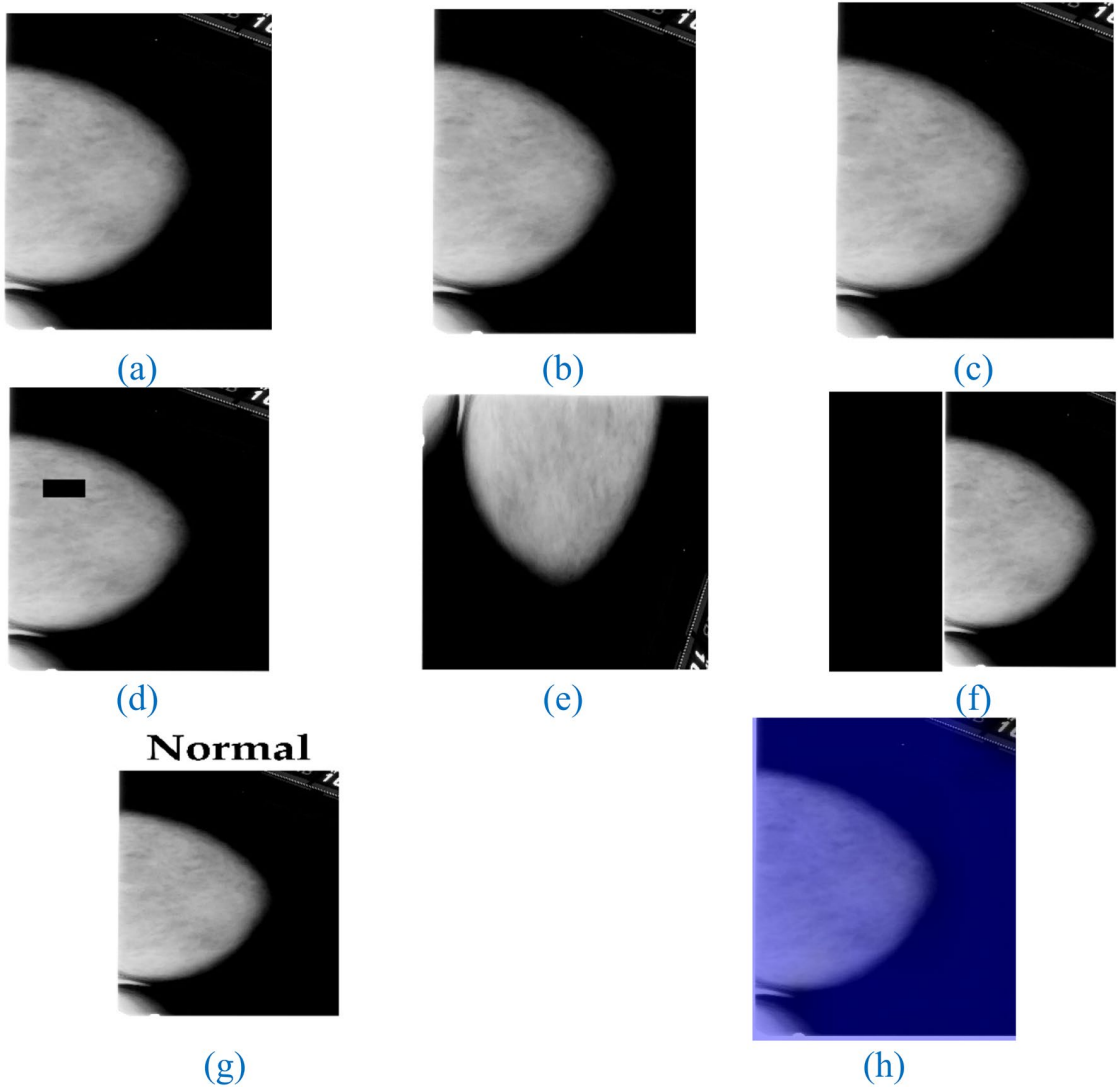
Fuzzy RS-Net output visualization corresponding to an normal image from the CBIS-DDSM dataset, (**a**) input, (**b**) pre-processed, (**c**) segmented, (**d**) random-erased, (**e**) rotation-augmented, (**f**) shifting-augmented images, (**g**) detected output, and (**h**) heatmap.

The images generated by the Fuzzy RS-Net corresponding to a normal CBIS-DDSM dataset image are shown in Fig. 11. Figure 11(a) and Fig. 11(b) indicate the input and pre-processed images. The segmented and random-erased images are presented in Fig. 11(c) and Fig. 11(d). Figure 11(e), Fig. 11(f) and Fig. 11(g) depict the rotation-augmented image, shifting-augmented image, and detected output image, respectively, and Fig. 11(h) illustrates the heatmap of the final output.

### Comparative methods

The Fuzzy RS-Net efficiency is examined by comparatively evaluating it with different techniques, such as Two-stage DL^6^, DFeBCD^5^, KM + + CSO^7^, TTCNN^16^, DenseNet121-based Extreme Learning Machine Model (ELM)^25^, Attention-Augmented Residual CNN (A2Res-CNN)^18^, and EfficientViewNet^19^.

### Comparative techniques

The evaluation of Fuzzy RS-Net against traditional methods on the MIAS and DDSM datasets is discussed in detail below.

#### With the MIAS dataset

**Fig. 12:**
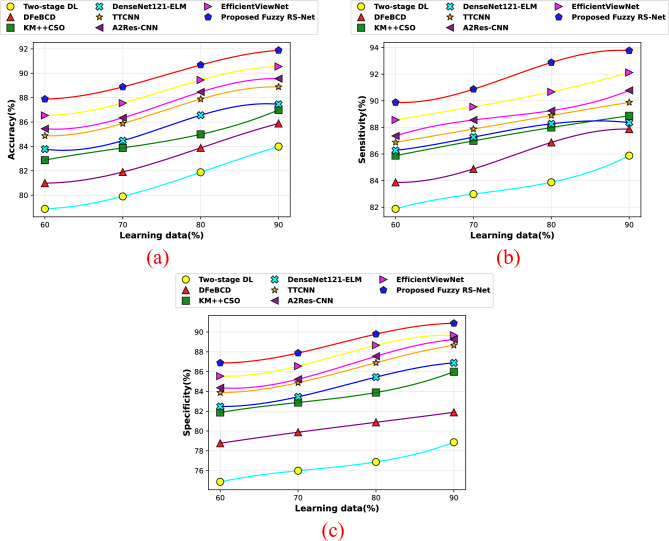
Appraisal of the Fuzzy RS-Net based on the MIAS dataset in terms of (**a**) accuracy, (**b**) sensitivity, and (**c**) specificity.

The performance results of Fuzzy RS-Net on the MIAS dataset are depicted in Fig. 12. The evaluation of Fuzzy RS-Net with respect to accuracy is shown in Fig. 12(a). The Fuzzy RS-Net, with the 60% learning data, measured an accuracy of 87.877%, and the existing methodologies namely Two-stage DL, DFeBCD, KM + + CSO, DenseNet121-ELM, TTCNN, A2Res-CNN, and EfficientViewNet, measured an accuracy of 78.877%, 80.989%, 82.879%, 83.777%, 84.868%, 85.444%, and 86.540%. The accuracy acquired by Fuzzy RS-Net is 3.42% better than the Two-stage DL. Figure 12(b) depicts the evaluation of Fuzzy RS-Net in terms of sensitivity. In this case, the sensitivity figured by the traditional approaches, such as Two-stage DL, DFeBCD, KM + + CSO, DenseNet121-ELM, TTCNN, A2Res-CNN, and EfficientViewNet, is 82.987%, 84.876%, 86.987%, 87.268%, 87.876%, 88.555%, and 89.545%, for the learning data of 70%, whereas the devised Fuzzy RS-Net achieved the sensitivity of 90.868%. The sensitivity of the Fuzzy RS-Net is augmented by 4.28% than the KM + + CSO. Figure 12(c) demonstrates the evaluation of the Fuzzy RS-Net regarding the specificity. The specificity recorded for the 80%, learning data by the Fuzzy RS-Net is 89.787%, whereas the value attained by the classical methods, namely Two-stage DL is 76.877%, DFeBCD is 80.879%, KM + + CSO is 83.887%, DenseNet121-ELM is 85.445%, TTCNN is 86.898%, A2Res-CNN is 87.555%, and EfficientViewNet is 88.860%. The presented Fuzzy RS-Net obtained a higher specificity by 3.45% than the KM + + CSO.

#### With the DDSM dataset

**Fig. 13:**
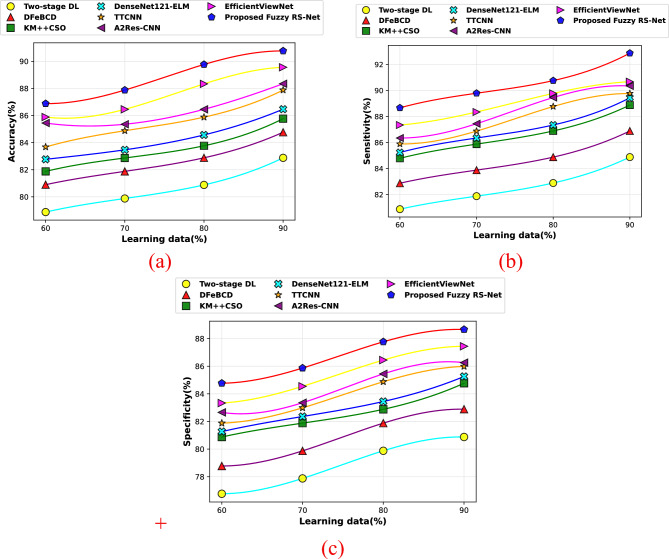
Assessment of the Fuzzy RS-Net by employing the DDSM Dataset concerning (**a**) accuracy, (**b**) sensitivity, and (**c**) specificity.

The assessment results of Fuzzy RS-Net on the DDSM dataset are illustrated in Fig. 13. The assessment of Fuzzy RS-Net with respect to accuracy is presented in Fig. 13(a). The accuracy acquired, with 60% of learning data, by the Fuzzy RS-Net is 86.877%, the accuracy gained by the prevailing methodologies is 78.876% for Two-stage DL, 80.898% for DFeBCD, 81.877% for KM + + CSO, 82.768% for DenseNet121-ELM, 83.677% for TTCNN, 85.445% for A2Res-CNN, and 85.888% for EfficientViewNet. The introduced Fuzzy RS-Net gained a performance improvement of 4.33% over the KM + + CSO. Figure 13(b) shows the evaluation of Fuzzy RS-Net with respect to the sensitivity metric. The Fuzzy RS-Net obtained a sensitivity of 89.788% and other classical models, like Two-stage DL, DFeBCD, KM + + CSO, DenseNet121-ELM, TTCNN, A2Res-CNN, and EfficientViewNet, recorded a sensitivity of 81.877%, 83.877%, 85.877%, 86.345%, 86.876%, 87.445%, and 88.345%, for the learning data of 70%. The sensitivity of the designed Fuzzy RS-Net is 3.34% better than that of the TTCNN. The appraisal of the Fuzzy RS-Net in view of specificity is embodied in Fig. 13(c). The Fuzzy RS-Net measured a specificity of 87.767%, when the learning data of 80%, and the classical existing schemes, namely Two-stage DL, DFeBCD, KM + + CSO, DenseNet121-ELM, TTCNN, A2Res-CNN, and EfficientViewNet, obtained a specificity of 79.877%, 81.879%, 82.879%, 83.444%, 84.876%, 85.444%, and 86.444%. Fuzzy RS-Net demonstrates a 3.41% higher specificity than the DFeBCD method.

#### With the CBIS-DDSM dataset

**Fig. 14:**
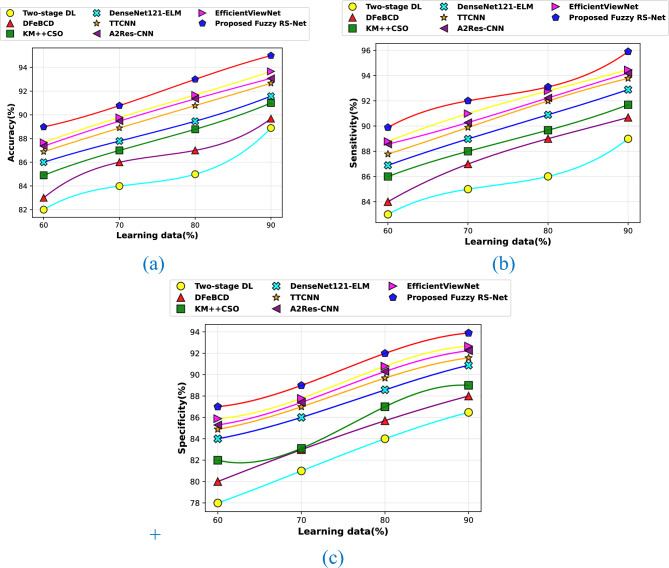
Evaluation of the Fuzzy RS-Net by using the CBIS-DDSM Dataset regarding (**a**) accuracy, (**b**) sensitivity, and (**c**) specificity.

In Fig. 14, the assessment of the CBIS-DDSM dataset for the learning data is presented. Figure 14(a) depicts the evaluation based on accuracy. For 60% of the learning data, the accuracy reached by the existing models are 81.999%, 82.999%, 84.900%, 85.999%, 86.888%, 87.357%, and 87.668%, while the proposed Fuzzy RS-Net approach achieves the maximum accuracy of 88.980%. Figure 14(b) depicts the model’s performance based on the sensitivity. The introduced Fuzzy RS-Net model and traditional techniques obtain the sensitivity of 91.999%, 84.999%, 86.988%, 87.999%, 88.980%, 89.888%, 90.279%, and 90.988%, for 70% of the learning data. Similarly, Fig. 14(c) shows the specificity analysis. For 80% of the learning data, the developed Fuzzy RS-Net model achieves the extreme specificity of 93.889%; meanwhile, the classical techniques reach the specificity of 86.467%, 87.999%, 88.999%, 90.878%, 91.567%, 92.236%, and 92.668%, respectively.

### Performance examination

The learning performance of the Fuzzy RS-Net with respect to the DDSM and MIAS datasets is discussed in this section.

#### With the MIAS dataset

**Fig. 15:**
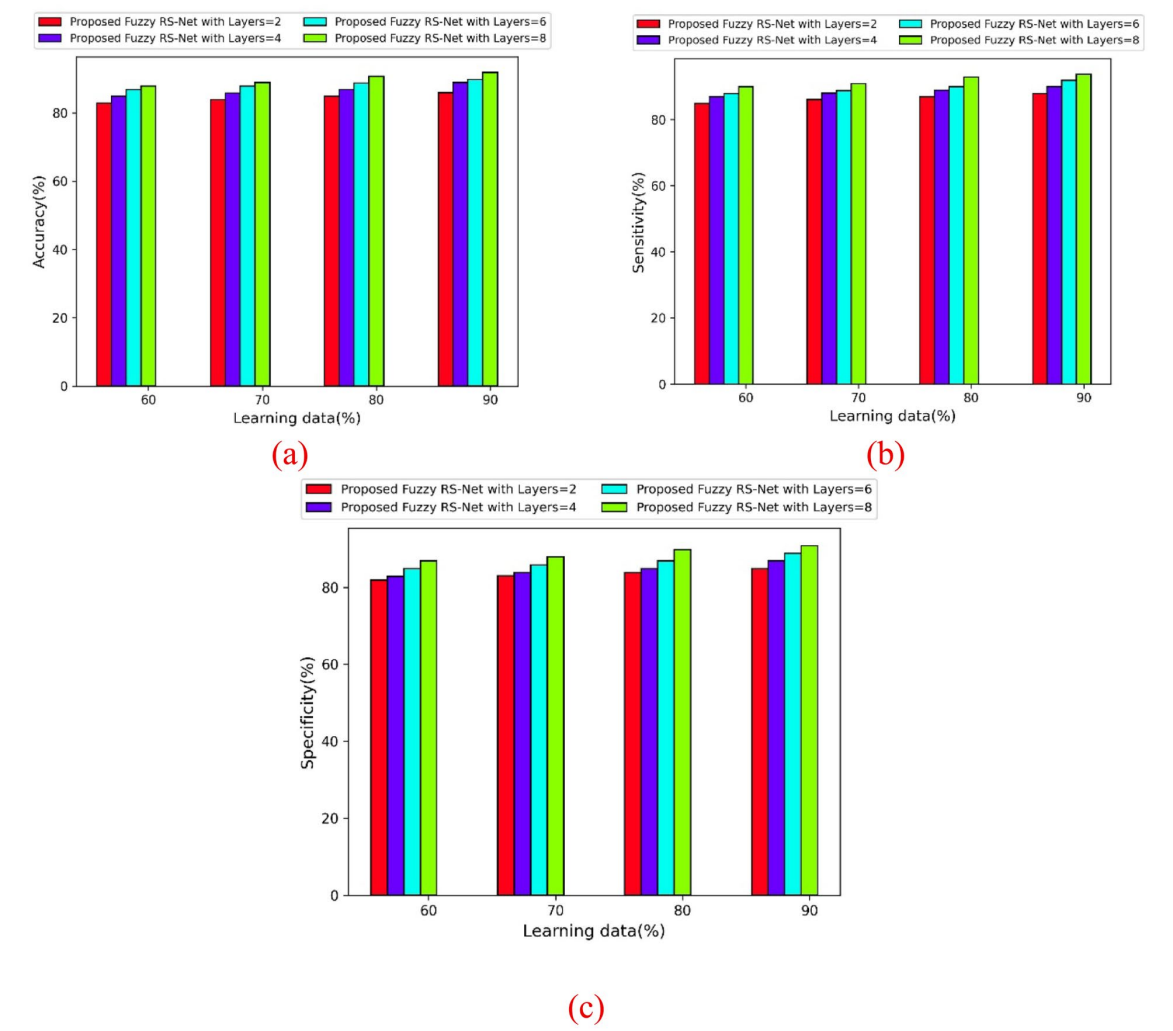
Performance investigation of the Fuzzy RS-Net about the MIAS dataset (**a**) accuracy, (**b**) sensitivity, and (**c**) specificity.

The performance of Fuzzy RS-Net on the MIAS dataset is illustrated in Fig. 15. The performance of Fuzzy RS-Net in terms of accuracy is presented in Fig. 15(a). The Fuzzy RS-Net, with the 60% learning data, recorded an accuracy of 82.876%, 84.870%, 86.765%, and 87.877% for 2, 4, 6, and 8 layers. Figure 15(b) depicts the sensitivity-based performance of Fuzzy RS-Net. At the learning data of 70%, the sensitivity of the Fuzzy RS-Net with 2, 4, 6, and 8 layers is 85.987%, 87.988%, 88.788%, and 90.868%. The efficiency of the Fuzzy RS-Net using the specificity is introduced in Fig. 15(c). The specificity figured by the Fuzzy RS-Net with 2, 4, 6, and 8 layers is 83.877%, 84.879%, 86.877%, and 89.787% at 80% of learning data.

#### With DDSM dataset

**Fig. 16:**
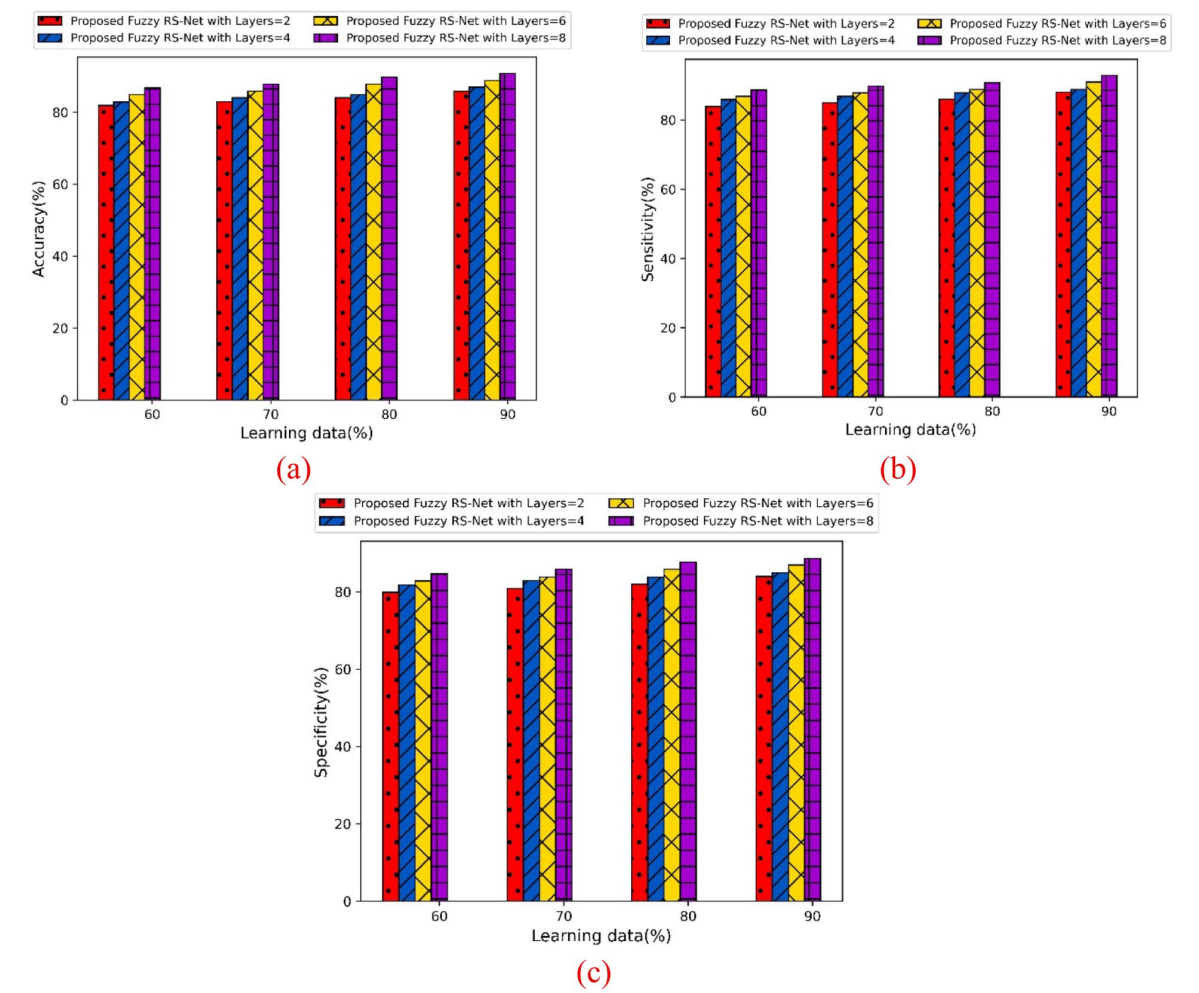
Performance’s investigation of the Fuzzy RS-Net in terms of learning data by exploiting the DDSM dataset: (**a**) accuracy, (**b**) sensitivity, and (**c**) specificity.

The performance of Fuzzy RS-Net on the DDSM dataset is illustrated in Fig. 16. Figure 16(a) highlights the accuracy evaluation. The accuracy of the Fuzzy RS-Net, at 60% of learning data, is 81.877%, 82.876%, 84.878%, and 86.877% for 2, 4, 6, and 8 layers. Figure 16(b) shows the assessment of Fuzzy RS-Net with respect to sensitivity. The Fuzzy RS-Net recorded sensitivity of 84.987%, 86.870%, 87.877%, and 89.788% with the learning data of 70% for 2, 4, 6, and 8 layers. The performance of Fuzzy RS-Net in terms of specificity is presented in Fig. 16(c). Here, with 80% learning data, the specificity achieved by the Fuzzy RS-Net for 2, 4, 6, and 8 layers is 81.987%, 83.877%, 85.876%, and 87.767%.

### Analysis of cross-validation

**Fig. 17:**
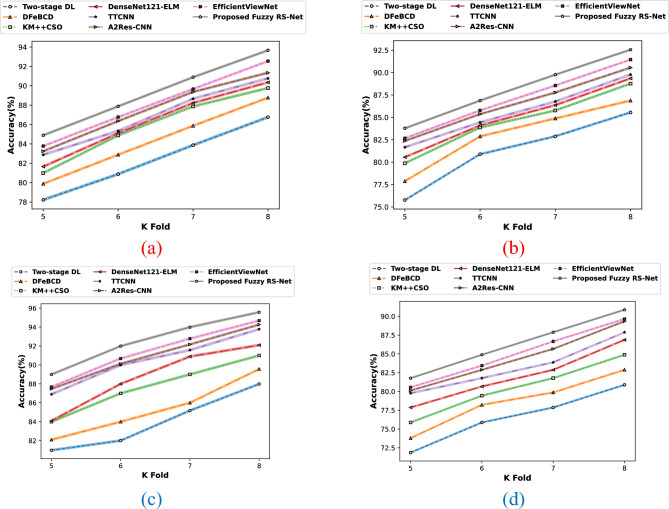
Cross-validation analysis for (**a**) MIAS dataset, (**b**) DDSM dataset, (**c**) CBIS-DDSM dataset, and (**d**) Cross-dataset.

To assess the model’s performance and ability to generalize, the dataset is divided into several folds as part of the cross-validation process. Training occurs on selected folds while testing is performed on the others, and the procedure is repeated to achieve a reliable evaluation. The cross-validation analysis is illustrated in Fig. 17, for the MIAS dataset, the DDSM dataset, the CBIS-DDSM dataset, and the cross-dataset, by comparing the existing models, such as Two-stage DL, DFeBCD, KM + + CSO, DenseNet121-ELM, TTCNN, A2Res-CNN, and EfficientViewNet, with the developed Fuzzy RS-Net approach, by varying accuracy and K-fold. In Fig. 17(a), the cross-validation for the MIAS dataset is demonstrated. For the K-fold of 8, the traditional models reach the accuracy of 86.760%, 88.770%, 89.767%, 90.357%, 90.766%, 91.347%, and 92.546%, while the introduced Fuzzy RS-Net technique achieves an accuracy of 93.665%. Figure 17(b) presents the cross-validation for the DDSM dataset. The classical and the proposed Fuzzy RS-Net approaches obtain an accuracy of 85.554%, 86.878%, 88.770%, 89.356%, 89.777%, 90.555%, 91.450%, and 92.554%, respectively, for a K-fold of 8. In Fig. 17(c), the evaluation of the CBIS-DDSM dataset is shown. When the K-fold is 8, the developed Fuzzy RS-Net model and the traditional techniques reach an accuracy of 95.568%, 87.990%, 89.568%, 90.999%, 92.091%, 93.778%, 94.258%, and 94.678%, respectively. The cross-dataset is shown in Fig. 17(d). For 8 K-fold, the proposed Fuzzy RS-Net model achieves an accuracy of 90.878%; meanwhile, the existing techniques reach an accuracy of 80.887%, 82.889%, 84.887%, 86.877%, 87.879%, 89.346%, and 89.656%. The proposed Fuzzy RS-Net model demonstrates superior performance compared to existing methods across all datasets. It achieves higher accuracy and consistency in cross-validation, indicating strong generalization capability. By incorporating fuzzy logic with deep learning, the model achieves better feature representation and classification, providing a robust and efficient solution for breast cancer detection.

### ROC curve

**Fig. 18:**
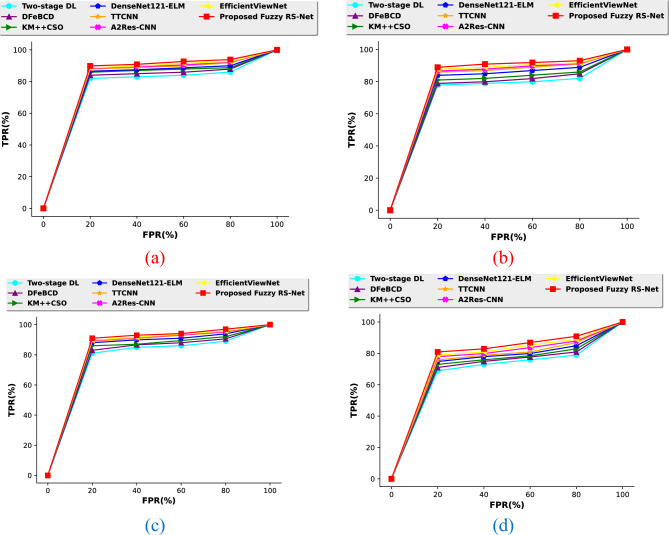
ROC curve for the Fuzzy RS-Net regarding the (**a**) MIAS dataset, (**b**) DDSM dataset, (**c**) CBIS-DDSM dataset, and (**d**) Cross-dataset.

Figure 18 illustrates the ROC curve for the Fuzzy RS-Net regarding the DDSM, MIAS, and CBIS-DDSM datasets, and cross-dataset. Figure 18(a) represents the ROC-based investigation for the Fuzzy RS-Net for the MIAS dataset. At 80% of FPR, the Fuzzy RS-Net attained a TPR of 93.868%, and the traditional methodologies, like Two-stage DL, DFeBCD, KM + + CSO, DenseNet121-ELM, TTCNN, A2Res-CNN, and EfficientViewNet, attained a TPR of 85.987%, 87.879%, 88.766%, 89.988%, 91.879%, 92.367%, and 92.666%. Figure 18(b) depicts the ROC-based performance evaluation of Fuzzy RS-Net using the DDSM dataset. With the FPR of 80%, the proposed Fuzzy RS-Net achieved a TPR of 92.988%, and the value attained by other classical approaches, such as Two-stage DL is 81.987%, DFeBCD is 84.899%, KM + + CSO is 85.987%, DenseNet121-ELM is 88.837%, TTCNN is 90.888%, A2Res-CNN is 91.364%, and EfficientViewNet is 91.667%. The ROC performance on the CBIS-DDSM dataset is illustrated in Fig. 18(c). When the FPR reaches 80%, the proposed Fuzzy RS-Net model achieves a TPR of 96.988%, whereas the traditional approaches attain TPR values of 88.989%, 90.567%, 91.999%, 93.879%, 94.899%, 95.368%, and 95.778% for Two-stage DL, DFeBCD, KM + + CSO, DenseNet121-ELM, TTCNN, A2Res-CNN, and EfficientViewNet, respectively. The ROC performance under cross-validation is presented in Fig. 18(d). For 80% of the FPR, the TPR reached by the classical models, namely Two-stage DL, DFeBCD, KM + + CSO, DenseNet121-ELM, TTCNN, A2Res-CNN, and EfficientViewNet, is 78.870%, 80.888%, 82.888%, 84.870%, 86.877%, 87.870%, and 88.889%, while the developed Fuzzy RS-Net approach achieves the maximum FPR of 90.877%.

### Cross-dataset validation

**Fig. 19:**
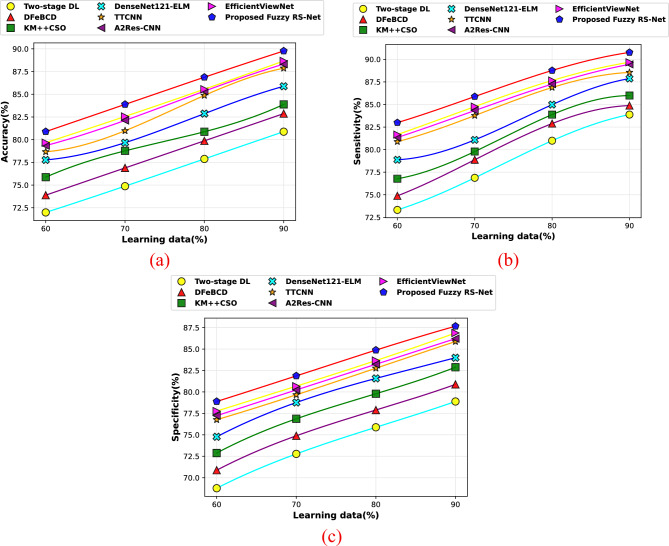
Cross-dataset validation for (**a**) Accuracy, (**b**) Sensitivity, and (**c**) Specificity.

To evaluate generalization across diverse data distributions, cross-dataset validation trains the model on one dataset and assesses its performance on a separate dataset. The MIAS dataset is used for training, while the DDSM dataset serves as the testing set in this analysis. Figure 19 presents the cross-dataset validation results, illustrating variations in accuracy, sensitivity, and specificity across the learning data. The performance of the proposed Fuzzy RS-Net model is compared with traditional approaches, including Two-stage DL, DFeBCD, KM + + CSO, DenseNet121-ELM, TTCNN, A2Res-CNN, and EfficientViewNet. Figure 19(a) depicts the accuracy obtained through cross-dataset evaluation. For 90% of the learning data, the proposed and the existing models reach an accuracy of 89.767%, 80.880%, 82.870%, 83.880%, 85.880%, 87.870%, 88.323%, and 88.666%. Figure 19(b) depicts the cross-dataset validation. The introduced approach obtains a sensitivity of 90.766%, while the traditional approaches reach a sensitivity of 83.880%, 84.880%, 85.990%, 87.870%, 88.550%, 89.444%, and 89.677%, respectively, for 90% the learning data. In Fig. 19(c), the specificity is illustrated. When the learning data is 90%, the classical and the proposed techniques achieve the specificity of 78.887%, 80.880%, 82.877%, 83.989%, 85.878%, 86.215%, 86.878%, and 87.666%. The proposed Fuzzy RS-Net model demonstrates strong generalization capability across different datasets. Compared with existing methods, it delivers higher accuracy, sensitivity, and specificity. Additionally, the model effectively handles variations in data distribution between training and testing datasets, ensuring consistent performance. As a result, the proposed approach offers dependable and robust performance in practical breast cancer detection applications.

### Ablation analysis

**Fig. 20:**
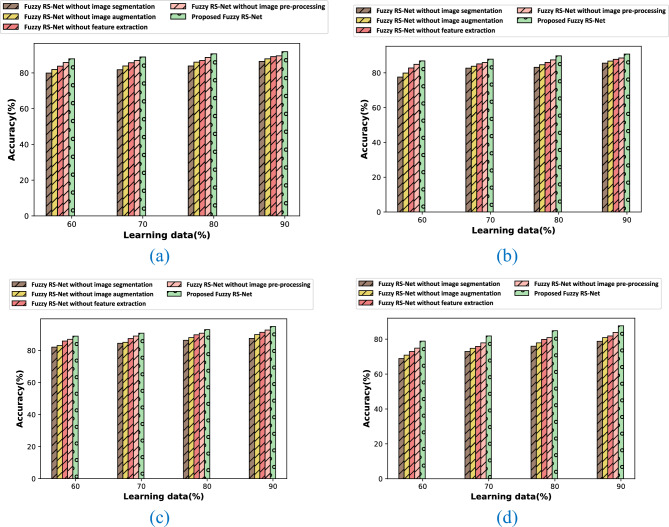
Ablation analysis for (**a**) MIAS dataset, (**b**) DDSM dataset, (**c**) CBIS-DDSM dataset, and (**d**) Cross-dataset.

The ablation analysis method assesses the importance of different model components by systematically removing or changing them and observing the effect on performance. Figure 20 shows the ablation analysis conducted on the MIAS dataset, DDSM dataset, the CBIS-DDSM dataset, and cross-dataset settings. A comparison is performed between the proposed Fuzzy RS-Net and its ablated versions, including those without pre-processing, segmentation, augmentation, and feature extraction, with respect to accuracy and training data variation. In Fig. 20(a), the ablation analysis for the MIAS dataset is illustrated. For 90% the learning data, the developed Fuzzy RS-Net approach achieves an accuracy of 91.876%, while the individual components reach the accuracy of 86.443%, 87.888%, 89.098%, and 89.555%. For the DDSM dataset, the ablation analysis is presented in Fig. 20(b). When the learning data is 90%, the separate modules and the introduced model obtain an accuracy of 85.545%, 86.767%, 87.777%, 88.555%, and 89.223%. In Fig. 20(c), the ablation for the CBIS-DDSM dataset is demonstrated. For 90% of the learning data, the distinct modules reach the accuracy of 87.577%, 89.910%, 91.346%, and 92.778%, while the developed approach achieves the accuracy of 94.999%. The cross-dataset ablation analysis is illustrated in Fig. 20(d). The proposed Fuzzy RS-Net and its distinct components achieve an accuracy of 87.666%, 78.778%, 80.877%, 81.877%, and 83.888%, for 90% of the learning data. The outcomes of the ablation analysis reveal the effectiveness of all Fuzzy RS-Net components, as the full model consistently surpasses the reduced versions in terms of accuracy. The contributions of pre-processing, segmentation, augmentation, and feature extraction collectively improve performance, making the combined architecture highly efficient, robust, and dependable for breast cancer detection.

### Confusion matrix

**Fig. 21:**
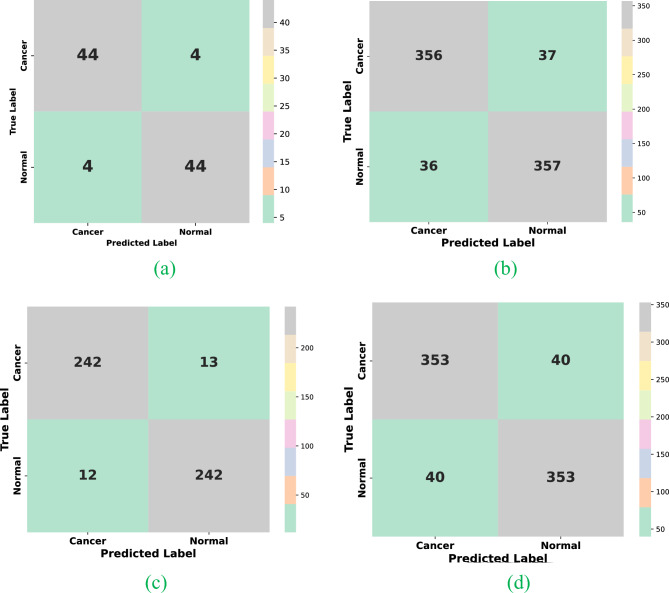
Confusion matrix for (**a**) MIAS dataset, (**b**) DDSM dataset, (**c**) CBIS-DDSM dataset, and (**d**) Cross-dataset.

The confusion matrix provides an evaluation of classification results by comparing the ground-truth labels with the predicted labels. It reports true positives, true negatives, false positives, and false negatives, enabling the computation of performance measures including accuracy, sensitivity, and specificity. The confusion matrix results for the MIAS dataset, DDSM dataset, the CBIS-DDSM dataset, and cross-dataset are illustrated in Fig. 21, showing the comparison between true and predicted labels for normal and cancerous cases. In Fig. 21(a), the confusion matrix for the MIAS dataset is shown. It illustrates that the model correctly classifies 44 cancer images as cancer and 44 normal images as normal. Meanwhile, 4 cancer images are incorrectly predicted as normal, and 4 normal images are incorrectly predicted as cancer. The confusion matrix for the DDSM dataset is illustrated in Fig. 21(b). The model correctly predicts 356 cancer images and 357 normal images. At the same time, 37 cancer images are misclassified as normal, and 36 normal images are misclassified as cancer. Figure 21(c) presents the confusion matrix obtained for the CBIS-DDSM dataset. As observed, the proposed model accurately identifies 242 cancer cases and 242 normal cases. However, 13 cancer cases are incorrectly predicted as normal, while 12 normal cases are incorrectly predicted as cancer. The higher number of correctly classified samples compared to misclassified samples demonstrates the robustness and reliability of the proposed breast cancer detection framework. Figure 21(d) presents the confusion matrix for the cross-dataset evaluation. Here, the model correctly identifies 353 cancer images and 353 normal images. There are 40 cancer images predicted as normal and 40 normal images predicted as cancer. The confusion matrix results collectively highlight the robustness and reliability of the proposed Fuzzy RS-Net model. It shows high accuracy and sensitivity across different datasets. The model handles both small and large datasets effectively, demonstrates strong generalization capability, and provides reliable classification results, which are crucial for early breast cancer detection.

### Computational time and memory analysis

The model’s efficiency is assessed through computational time and memory usage analysis, which helps determine its scalability and practicality for real-time deployment. Table 4 presents the analysis of computational time and memory usage for the MIAS, DDSM, CBIS-DDSM, and cross-datasets for a single image. The performance of the proposed Fuzzy RS-Net model is compared with traditional models, including Two-stage DL, DFeBCD, KM + + CSO, DenseNet121-ELM, TTCNN, A2Res-CNN, and EfficientViewNet. For the MIAS, DDSM, CBIS-DDSM, and cross-datasets, the proposed Fuzzy RS-Net approach achieves lower computational times of 18.887 s, 23.766 s, 29.988 s, and 26.766 s, respectively, compared to existing techniques. Furthermore, the proposed Fuzzy RS-Net model utilizes minimal memory of 8.5 MB, 9.5 MB, 38.7 MB, and 10.3 MB for the MIAS, DDSM, CBIS-DDSM, and cross-datasets, respectively, which is lower than that of the conventional models. The performance variation observed in cross-dataset evaluation is primarily due to domain shift between MIAS and DDSM datasets. MIAS images are relatively lower in resolution and exhibit simpler structural patterns, whereas DDSM images contain higher resolution mammograms with greater anatomical complexity and variations in imaging conditions. In addition, differences in data acquisition protocols, scanner settings, and patient populations contribute to distribution mismatch across datasets. This domain discrepancy leads to slight variations in feature representation, which explains the minor performance fluctuations in cross-dataset testing. However, the proposed Fuzzy RS-Net maintains strong generalization capability due to its robust preprocessing using wavelet filtering, deep feature extraction through DRN, efficient representation learning via ShuffleNet, and uncertainty handling using fuzzy logic. These components collectively enhance domain invariance and enable the model to adapt effectively across different datasets, demonstrating its robustness for real-world breast cancer detection scenarios. Furthermore, the proposed Fuzzy RS-Net demonstrates superior efficiency in terms of both computational time and memory usage, as it requires significantly less processing time compared to existing models, making it suitable for real-time applications. Its low memory consumption also ensures better scalability and supports deployment on resource-constrained systems. Overall, the proposed framework offers an efficient, fast, lightweight, and highly generalizable solution for breast cancer detection across diverse datasets.

**Table 4:** Analysis of computational time and memory.

Dataset	Existing models							Proposed fuzzy RS-net
	**Two-stage DL**	**DFeBCD**	**KM + + CSO**	**DenseNet121-ELM**	**TTCNN**	**A2Res-CNN**	**EfficientViewNet**	
*Computational time (sec)*								
MIAS	42.877	39.870	37.877	33.777	29.777	25.777	21.878	***18.887***
DDSM	48.870	44.877	39.770	36.878	33.766	29.766	26.877	23.766
CBIS-DDSM	55.988	49.999	47.990	45.100	42.999	39.999	35.999	29.988
Cross dataset	51.877	46.776	41.777	39.770	35.770	30.766	28.770	26.766
*Memory (MB)*								
MIAS	18.7	16.7	15.9	15.6	13.6	11.5	9.5	***8.5***
DDSM	19.5	18.5	16.9	16.3	15.4	11.9	10.5	9.5
CBIS-DDSM	47.2	46.6	44.8	44.6	43.5	41.5	40.7	38.7
Cross dataset	21.5	19.5	17.5	16.9	16.3	13.5	10.8	10.3

The computational time and memory usage for the ablation study are presented in Table 5 by comparing individual variants of the model, such as Fuzzy RS-Net without pre-processing, without segmentation, without augmentation, and without feature extraction, against the complete proposed Fuzzy RS-Net framework. For the MIAS dataset, the computational time for the ablated configurations is 10.988 s, 12.990 s, 13.999 s, and 12.799 s, respectively, whereas the complete Fuzzy RS-Net achieves a computational time of 18.887 s. Similarly, the memory consumption for the different modules and the proposed model is 23.2 MB, 24.6 MB, 25.8 MB, 27.7 MB, and 28.5 MB, respectively. Overall, the ablation results demonstrate the trade-off between model components and computational cost, highlighting that the proposed framework maintains efficient memory usage while integrating all essential modules. These findings confirm the practical efficiency and optimized design of the Fuzzy RS-Net for breast cancer detection.

**Table 5:** Computational time and memory for ablation.

Dataset	Individual components				Proposed fuzzy RS-net
	**Fuzzy RS-Net without image segmentation**	**Fuzzy RS-Net without image augmentation**	**Fuzzy RS-Net without feature extraction**	**Fuzzy RS-Net without image pre-processing**	
*Computational time (sec)*					
MIAS	10.988	12.990	13.999	12.779	**18.887**
DDSM	13.888	14.999	15.999	16.999	23.766
CBIS-DDSM	15.998	17.999	20.990	23.999	26.766
Cross dataset	18.999	22.999	25.009	27.999	29.988
*Memory (MB)*					
MIAS	23.2	24.6	25.8	27.7	**28.5**
DDSM	24.7	25.8	26.7	28.6	29.5
CBIS-DDSM	26.2	27.7	28.5	29.6	30.3
Cross dataset	30.7	33.7	35.7	37.6	38.7

### T-test analysis

Statistical significance is analyzed using paired t-tests applied to the accuracy, sensitivity, and specificity results of the proposed model and existing baseline methods. Under a tenfold cross-validation framework, the performance of the proposed method is evaluated against classical models across the same data folds. Statistical significance is determined using a threshold p-value of 0.05, with paired t-tests applied to accuracy, sensitivity, and specificity to evaluate the performance improvements of the proposed method over existing approaches. The t-test analysis is conducted over fivefold cross-validation for the MIAS, DDSM, CBIS-DDSM, and cross-datasets, and the results are shown in Table 6. The analysis indicates that the proposed method consistently outperforms all baseline models, including Two-stage DL, DFeBCD, KM + + CSO, DenseNet121-ELM, TTCNN, A2Res-CNN, and EfficientViewNet, across the three-evaluation metrics, namely accuracy, sensitivity, and specificity, for MIAS, DDSM, CBIS-DDSM, and cross-datasets. For the MIAS dataset, all comparisons show statistically significant results with p < 0.05. The proposed method achieves higher accuracy compared to Two-stage DL (t = 3.28, p = 0.02), DFeBCD (t = 2.98, p = 0.02), KM + + CSO (t = 2.60, p = 0.02), DenseNet121-ELM (t = 2.39, p = 0.03), TTCNN (t = 2.18, p = 0.03), A2Res-CNN (t = 2.18, p = 0.03), and EfficientViewNet (t = 1.80, p = 0.04). Improvements in sensitivity and specificity are also statistically significant. Results from the DDSM dataset validation confirm that the proposed method consistently outperforms the baseline models across all evaluation criteria. In comparison with Two-stage DL, the t-statistic values are 2.95, 3.16, and 2.77 for the three metrics, with a corresponding p-value of 0.02. Statistically significant differences are also observed against DFeBCD (p = 0.02 and 0.03), KM + + CSO (p = 0.03), DenseNet121-ELM (p = 0.03 and 0.04), TTCNN (p = 0.03 and 0.04), A2Res-CNN (p = 0.04), and EfficientViewNet (p = 0.04 and 0.05). The proposed method shows superior performance over all baseline approaches on the DDSM dataset across all measured metrics. When compared with Two-stage DL, the t-statistics are 3.49, 3.70, and 3.31, for the three metrics, and the p-value is 0.02. Significant statistical improvements are further noted against DFeBCD (p = 0.02 and 0.03), KM + + CSO (p = 0.02 and 0.03), DenseNet121-ELM (p = 0.03), TTCNN (p = 0.03 and 0.04), A2Res-CNN (p = 0.03 and 0.04), and EfficientViewNet (p = 0.04 and 0.05). On cross-dataset evaluation, the proposed method shows consistent superiority over all baseline models across all metrics. When compared with Two-stage DL, the t-statistic values are 3.60, 3.81, and 3.42 for the three metrics, with p-values of 0.01 and 0.02. Significant statistical differences are also observed for DFeBCD (p = 0.02), KM + + CSO (p = 0.02 and 0.03), DenseNet121-ELM (p = 0.02 and 0.03), TTCNN (p = 0.03), A2Res-CNN (p = 0.03 and 0.04), and EfficientViewNet (p = 0.03 and 0.04). Additional validation using cross-dataset testing demonstrates that the proposed method consistently outperforms every baseline model across all evaluated metrics. For instance, in comparison with Two-stage DL, the t-statistic values are 3.60, 3.81, and 3.42 for the three metrics, with p-values of 0.01 and 0.02. Likewise, significant statistical differences are identified when compared with DFeBCD (p = 0.02), KM + + CSO (p = 0.02 and 0.03), DenseNet121-ELM (p = 0.02 and 0.03), TTCNN (p = 0.03), A2Res-CNN (p = 0.03 and 0.04), and EfficientViewNet (p = 0.03 and 0.04). The consistent and statistically significant outcomes across datasets validate the effectiveness of the proposed method, demonstrating its robustness and superior performance compared to existing approaches.

**Table 6:** T-test analysis.

Proposed model	Existing models	T-statistics			P-value		
		Accuracy	Sensitivity	Specificity	Accuracy	Sensitivity	Specificity
MIAS dataset							
***Proposed fuzzy RS-Net***	*Two-stage DL*	3.28	3.49	3.10	0.02	0.01	0.02
	*DFeBCD*	2.98	3.28	2.79	0.02	0.02	0.02
	*KM* + + *CSO*	2.60	2.99	2.38	0.02	0.02	0.03
	*DenseNet121-ELM*	2.39	2.69	2.18	0.03	0.03	0.03
	*TTCNN*	2.18	2.38	1.88	0.03	0.03	0.04
	*A2Res-CNN*	1.88	2.10	1.58	0.04	0.03	0.04
	*EfficientViewNet*	1.80	1.89	1.51	0.04	0.03	0.04
DDSM dataset							
***Proposed fuzzy RS-net***	*Two-stage DL*	2.95	3.16	2.77	0.02	0.02	0.02
	*DFeBCD*	2.65	2.95	2.46	0.03	0.02	0.03
	*KM* + + *CSO*	2.27	2.66	2.05	0.03	0.03	0.03
	*DenseNet121-ELM*	2.06	2.36	1.85	0.03	0.03	0.04
	*TTCNN*	1.85	2.05	1.55	0.04	0.03	0.04
	*A2Res-CNN*	1.55	1.77	1.25	0.04	0.04	0.04
	*EfficientViewNet*	1.47	1.56	1.18	0.04	0.04	0.05
CBIS-DDSM dataset							
***Proposed fuzzy RS-net***	*Two-stage DL*	3.49	3.70	3.31	0.02	0.02	0.02
	*DFeBCD*	3.19	3.49	3.00	0.02	0.02	0.03
	*KM* + + *CSO*	2.81	3.20	2.59	0.03	0.02	0.03
	*DenseNet121-ELM*	2.60	2.90	2.39	0.03	0.03	0.03
	*TTCNN*	2.39	2.59	2.09	0.04	0.03	0.04
	*A2Res-CNN*	2.09	2.31	1.79	0.04	0.03	0.04
	*EfficientViewNet*	2.01	2.10	1.72	0.04	0.04	0.05
Cross-dataset							
***Proposed fuzzy RS-net***	*Two-stage DL*	3.60	3.81	3.42	0.01	0.01	0.02
	*DFeBCD*	3.30	3.60	3.12	0.02	0.02	0.02
	*KM* + + *CSO*	2.92	3.31	2.70	0.02	0.02	0.03
	*DenseNet121-ELM*	2.71	3.01	2.50	0.03	0.02	0.03
	*TTCNN*	2.50	2.70	2.20	0.03	0.03	0.03
	*A2Res-CNN*	2.20	2.42	1.90	0.03	0.03	0.04
	*EfficientViewNet*	2.12	2.21	1.83	0.04	0.03	0.04

### Wilcoxon signed-rank test

The statistical difference between the proposed method and baseline models is assessed through the Wilcoxon signed-rank test based on their respective tenfold cross-validation performance results. The evaluation is conducted independently for three metrics, accuracy, sensitivity, and specificity. For each metric, the proposed method is compared with each baseline method, such as Two-stage DL, DFeBCD, KM + + CSO, DenseNet121-ELM, TTCNN, A2Res-CNN, and EfficientViewNet on a pairwise basis using fold-wise results, ensuring proper paired statistical testing. In order to handle multiple pairwise comparisons, p-values are adjusted through the Holm method, and significance is determined when the adjusted p-value is below 0.05. Table 7 summarizes the Wilcoxon signed-rank statistics, original and adjusted p-values for all comparisons. For the MIAS dataset, the proposed method achieved significantly higher accuracy, sensitivity, and specificity than Two-stage DL (*p* = 0.04) after Holm correction. On the DDSM dataset, the introduced method shows significantly improved accuracy, sensitivity, and specificity over DFeBCD after Holm correction, with p = 0.03. In the CBIS-DDSM dataset, the proposed approach attains significantly better accuracy, sensitivity, and specificity than KM + + CSO, with statistical significance confirmed after Holm correction (p = 0.03). The proposed method on the cross-dataset demonstrates significantly higher accuracy, sensitivity, and specificity than DenseNet121-ELM, with statistical significance retained after Holm correction (p = 0.03). Overall, the Wilcoxon signed-rank test results confirm that the proposed Fuzzy RS-Net consistently achieves statistically significant improvements over all baseline models across MIAS, DDSM, CBIS-DDSM, and cross-dataset evaluations. The use of Holm correction further strengthens the reliability of these findings by controlling for multiple comparisons. The obtained results demonstrate the effectiveness, robustness, and capability of the proposed method to generalize for breast cancer detection.

**Table 7:** Wilcoxon signed-rank test.

Proposed model	Existing models	Wilcoxon-statics			P-value			Adjusted- P-value (Holm–Bonferroni)		
		**Acc**	**Sen**	**Spe**	**Acc**	**Sen**	**Spe**	**Acc**	**Sen**	**Spe**
**MIAS dataset**										
***Proposed fuzzy RS-Net***	*Two-stage DL*	3.57	3.19	3.99	0.04	0.04	0.04	0.04	0.04	0.04
	*DFeBCD*	3.01	2.78	3.58	0.03	0.03	0.04	0.04	0.03	0.04
	*KM* + + *CSO*	2.79	2.58	3.37	0.03	0.03	0.03	0.03	0.03	0.04
	*DenseNet121-ELM*	2.48	2.38	2.79	0.03	0.03	0.03	0.03	0.03	0.03
	*TTCNN*	2.18	1.99	2.48	0.02	0.02	0.03	0.03	0.03	0.03
	*A2Res-CNN*	1.98	1.91	2.10	0.02	0.02	0.03	0.02	0.02	0.03
	*EfficientViewNet*	1.82	1.77	1.99	0.02	0.02	0.02	0.02	0.02	0.03
**DDSM dataset**										
***Proposed fuzzy RS-Net***	*Two-stage DL*	3.23	2.85	3.65	0.03	0.03	0.03	0.04	0.03	0.04
	*DFeBCD*	2.67	2.44	3.24	0.03	0.03	0.03	0.03	0.03	0.03
	*KM* + + *CSO*	2.45	2.24	3.03	0.03	0.03	0.03	0.03	0.03	0.03
	*DenseNet121-ELM*	2.14	2.04	2.45	0.02	0.02	0.03	0.03	0.03	0.03
	*TTCNN*	1.84	1.65	2.14	0.02	0.02	0.02	0.02	0.02	0.03
	*A2Res-CNN*	1.64	1.57	1.76	0.02	0.02	0.02	0.02	0.02	0.02
	*EfficientViewNet*	1.48	1.43	1.65	0.02	0.01	0.02	0.02	0.02	0.02
**CBIS-DDSM dataset**										
***Proposed fuzzy RS-Net***	*Two-stage DL*	3.35	2.97	3.77	0.03	0.03	0.04	0.04	0.04	0.04
	*DFeBCD*	2.79	2.56	3.36	0.03	0.03	0.03	0.03	0.03	0.04
	*KM* + + *CSO*	2.57	2.36	3.15	0.03	0.03	0.03	0.03	0.03	0.03
	*DenseNet121-ELM*	2.26	2.16	2.57	0.03	0.02	0.03	0.03	0.03	0.03
	*TTCNN*	1.96	1.77	2.26	0.02	0.02	0.03	0.02	0.02	0.03
	*A2Res-CNN*	1.76	1.69	1.88	0.02	0.02	0.02	0.02	0.02	0.03
	*EfficientViewNet*	1.60	1.55	1.77	0.02	0.01	0.02	0.02	0.02	0.02
**Cross-dataset**										
***Proposed fuzzy RS-Net***	*Two-stage DL*	3.89	3.51	4.32	0.04	0.04	0.04	0.04	0.04	0.04
	*DFeBCD*	3.33	3.10	3.90	0.04	0.03	0.04	0.04	0.04	0.04
	*KM* + + *CSO*	3.12	2.91	3.69	0.03	0.03	0.04	0.04	0.03	0.04
	*DenseNet121-ELM*	2.80	2.70	3.11	0.03	0.03	0.03	0.03	0.03	0.03
	*TTCNN*	2.51	2.31	2.80	0.03	0.02	0.03	0.03	0.03	0.03
	*A2Res-CNN*	2.30	2.23	2.42	0.02	0.02	0.03	0.03	0.02	0.03
	*EfficientViewNet*	2.14	2.09	2.31	0.02	0.02	0.02	0.02	0.02	0.03

### Confidence intervals analysis

Confidence intervals provide a range of probable values for a performance metric at a certain confidence level, offering insight into the model’s stability and reliability. In Table 8, the analysis of confidence intervals is demonstrated for accuracy by comparing the classical approaches, such as Two-stage DL, DFeBCD, KM + + CSO, DenseNet121-ELM, TTCNN, A2Res-CNN, and EfficientViewNet, with the developed Fuzzy RS-Net model for the MIAS, DDSM, CBIS-DDSM, and cross-datasets. For the MIAS dataset, the proposed Fuzzy RS-Net achieves a confidence interval of (83.30, 95.36), which is higher than Two-stage DL (76.56, 88.31), DFeBCD (78.26, 90.44), KM + + CSO (79.78, 91.97), DenseNet121-ELM (80.30, 92.37), TTCNN (81.34, 92.47), A2Res-CNN (81.93, 93.22), and EfficientViewNet (82.19, 94.19). For the DDSM dataset, the proposed method achieves a confidence interval of (82.24, 94.25), which again outperforms Two-stage DL (74.69, 87.86), DFeBCD (76.98, 89.28), KM + + CSO (78.65, 90.50), DenseNet121-ELM (79.18, 91.00), TTCNN (80.18, 91.15), A2Res-CNN (80.97, 92.06), and EfficientViewNet (81.13, 93.10). On the CBIS-DDSM dataset, the introduced Fuzzy RS-Net obtains a confidence interval of (88.12, 97.15), outperforming Two-stage DL (78.98, 89.10), DFeBCD (80.34, 90.50), KM + + CSO (83.00, 92.50), DenseNet121-ELM (83.11, 94.43), TTCNN (85.96, 95.16), A2Res-CNN (86.39, 95.62), and EfficientViewNet (86.68, 96.23). Similarly, for the cross-dataset, the proposed Fuzzy RS-Net obtains a confidence interval of (80.12, 92.59), which is higher than all baseline methods, including Two-stage DL (70.61, 82.64), DFeBCD (72.65, 84.74), KM + + CSO (74.45, 86.55), DenseNet121-ELM (76.02, 88.12), TTCNN (77.81, 88.84), A2Res-CNN (78.23, 90.78), and EfficientViewNet (78.81, 91.35). The proposed Fuzzy RS-Net consistently achieves higher confidence interval ranges, indicating improved stability and reliability of performance. This demonstrates its robustness and effectiveness over existing baseline models across all datasets.

**Table 8:** Validation of confidence intervals.

Methods	MIAS	DDSM	CBIS-DDSM	Cross-dataset
Two-stage DL	(76.56, 88.31)	(74.69, 87.86)	(78.98, 89.10)	(70.61, 82.64)
DFeBCD	(78.26, 90.44)	(76.98, 89.28)	(80.34, 90.50)	(72.65, 84.74)
KM + + CSO	(79.78, 91.97)	(78.65, 90.50)	(83.00, 92.50)	(74.45, 86.55)
DenseNet121-ELM	(80.30, 92.37)	(79.18, 91.00)	(83.11, 94.43)	(76.02, 88.12)
TTCNN	(81.34, 92.47)	(80.18, 91.15)	(85.96, 95.16)	(77.81, 88.84)
A2Res-CNN	(81.93, 93.22)	(80.97, 92.06)	(86.39, 95.62)	(78.23, 90.78)
EfficientViewNet	(82.19, 94.19)	(81.13, 93.10)	(86.68, 96.23)	(78.81, 91.35)
***Proposed fuzzy RS-Net***	***(83.30, 95.36)***	***(82.24, 94.25)***	***(88.12, 97.15)***	***(80.12, 92.59)***

### Comparative discussion

The performance of Fuzzy RS-Net across various metrics with 90% training samples from the MIAS, DDSM, and CBIS-DDSM datasets is compared in Table 9. With 90% learning data, the devised Fuzzy RS-Net gained the highest accuracy, sensitivity, and specificity values of 94.999%, 95.899%, and 93.889%. At the same time, other traditional methods, like Two-stage DL, DFeBCD, KM + + CSO, DenseNet121-ELM, TTCNN, A2Res-CNN, and EfficientViewNet, acquired an accuracy of 88.888%, 89.678%, 90.999%, 91.578%, 92.668%, 93.080%, and 93.667%. The sensitivity obtained by the prevailing methodologies is 88.988%, 90.677%, 91.689%, 92.888%, 93.778%, 94.190%, and 94.456%, for the Two-stage DL, DFeBCD, KM + + CSO, DenseNet121-ELM, TTCNN, A2Res-CNN, and EfficientViewNet. At the learning data of 90%, the specificity attained by the Two-stage DL is 86.467%, DFeBCD is 87.999%, KM + + CSO is 88.999%, DenseNet121-ELM is 90.878%, TTCNN is 91.567%, A2Res-CNN is 92.236%, and EfficientViewNet is 92.668%. The fuzzy concept introduces uncertainty handling, which improves decision flexibility and enhances robustness in classifying ambiguous mammogram patterns. The DRN contributes effective deep feature learning and improves classification accuracy by capturing high-level representations. ShuffleNet reduces computational complexity and memory requirements, enabling a lightweight and scalable architecture suitable for efficient processing. By integrating these three components, the proposed Fuzzy RS-Net achieves a balanced trade-off between accuracy, efficiency, and robustness, resulting in superior performance for breast cancer detection.

**Table 9:** Comparative discussion.

*Dataset*	Measures	*Two-stage DL*	*DFeBCD*	*KM* + + *CSO*	*DenseNet121-ELM*	*TTCNN*	A2Res-CNN	EfficientViewNet	*Designed* *fuzzy RS-Net*
***MIAS dataset***	***Accuracy (%)***	83.988	85.877	86.987	87.455	88.876	89.555	90.550	91.876
	***Sensitivity (%)***	85.876	87.870	88.870	88.357	89.877	90.777	92.122	93.766
	***Specificity (%)***	78.867	81.887	85.990	86.888	88.665	89.235	89.677	90.877
***DDSM dataset***	***Accuracy (%)***	82.877	84.766	85.768	86.466	87.877	88.345	89.560	90.766
	***Sensitivity (%)***	84.879	86.887	88.876	89.434	89.767	90.344	90.677	92.868
	***Specificity (%)***	80.876	82.887	84.766	85.257	85.987	86.256	87.443	88.657
***CBIS-DDSM dataset***	***Accuracy (%)***	88.888	89.678	90.999	91.578	92.668	93.080	93.667	**94.999**
	***Sensitivity (%)***	88.988	90.677	91.689	92.888	93.778	94.190	94.456	**95.899**
	***Specificity (%)***	86.467	87.999	88.999	90.878	91.567	92.236	92.668	**93.889**

In clinical breast cancer screening, misclassification outcomes have different levels of risk, where false negatives are considered significantly more critical than false positives, as undetected malignant cases may delay treatment and adversely affect patient survival. Therefore, maximizing sensitivity is essential to ensure that potential cancer cases are not missed during screening. The proposed Fuzzy RS-Net prioritizes high sensitivity while maintaining balanced specificity, thereby reducing the likelihood of false negative predictions. This makes the model more suitable as a decision-support tool in real-world clinical settings, where early and reliable detection plays a crucial role in improving patient outcomes. However, the model is intended to assist radiologists rather than replace clinical diagnosis, ensuring safer and more reliable breast cancer screening support.

## Conclusion

A form of cancer affecting breast tissues, breast cancer requires early detection to increase survival chances and achieve more effective diagnoses. To resolve this issue, a new Fuzzy RS-Net model is developed to facilitate breast cancer detection. In the pre-processing stage, Wavelet domain filtering is applied to the mammogram image for noise reduction, after which O-SegNet performs segmentation of the affected regions. Image augmentation is applied using rotation, shifting, and random erasing, after which feature extraction is performed on the augmented images to obtain texture features, including Maximal Correlation Coefficient, ASM, Sum Entropy, PHoG, and WLBP. The detection of breast cancer is done by the Fuzzy RS-Net. The Fuzzy RS-Net is established by fusing the ShuffleNet, Fuzzy concept, and DRN. Furthermore, the designed Fuzzy RS-Net achieved the maximum value of accuracy at 94.999%, sensitivity at 95.899%, and specificity at 93.889%. Although the proposed Fuzzy RS-Net model demonstrates effective performance in breast cancer detection, certain limitations remain. The model is evaluated only on publicly available datasets, which may introduce dataset bias and limit its generalization to diverse clinical data. The real-world applicability in clinical settings is not yet validated, and the interpretability of the model decisions is limited due to the complexity of DL architectures. The improvement over certain baseline methods is relatively marginal in some cases. To overcome these limitations, future work will focus on validating the model using large-scale and diverse clinical datasets to improve generalization capability and incorporating advanced optimization strategies and improved feature fusion techniques to further enhance classification performance while maintaining efficiency. Further efforts will be made to integrate explainable artificial intelligence techniques to enhance interpretability of the model decisions.

## Data Availability

The data that support the findings of this study are openly available in the Mammographic Image Analysis Society (MIAS) database and Digital Database for Screening Mammography (DDSM)  dataset at https://www.mammoimage.org/databases , reference number^33^ and the CBIS-DDSM: Breast Cancer Image Dataset at  https://www.kaggle.com/datasets/awsaf49/cbis-ddsm-breast-cancer-image-dataset, reference number^34^.
